# Overexpression of human NR2B receptor subunit in LMAN causes stuttering and song sequence changes in adult zebra finches

**DOI:** 10.1038/s41598-017-00519-8

**Published:** 2017-04-21

**Authors:** Mukta Chakraborty, Liang-Fu Chen, Emma E. Fridel, Marguerita E. Klein, Rebecca A. Senft, Abhra Sarkar, Erich D. Jarvis

**Affiliations:** 1 Department of Neurobiology, Duke University Medical Center, Durham, NC 27710 USA; 2 Howard Hughes Medical Institute, Chevy Chase, MD USA; 3 Neurotransgenic Laboratory, Department of Neurobiology, Duke University, Durham, NC 27710 USA; 4 Department of Neurobiology, Harvard University, Cambridge, MA 02138 USA; 5 Department of Statistical Science, Duke University, Durham, NC 27710 USA; 6 Laboratory of Neurogenetics of Language, The Rockefeller University, New York, NY 10065 USA

## Abstract

Zebra finches (*Taeniopygia guttata*) learn to produce songs in a manner reminiscent of spoken language development in humans. One candidate gene implicated in influencing learning is the N-methyl-D-aspartate (NMDA) subtype 2B glutamate receptor (*NR2B)*. Consistent with this idea, *NR2B* levels are high in the song learning nucleus LMAN (lateral magnocellular nucleus of the anterior nidopallium) during juvenile vocal learning, and decreases to low levels in adults after learning is complete and the song becomes more stereotyped. To test for the role of *NR2B* in generating song plasticity, we manipulated *NR2B* expression in LMAN of adult male zebra finches by increasing its protein levels to those found in juvenile birds, using a lentivirus containing the full-length coding sequence of the human *NR2B* subunit. We found that increased *NR2B* expression in adult LMAN induced increases in song sequence diversity and slower song tempo more similar to juvenile songs, but also increased syllable repetitions similar to stuttering. We did not observe these effects in control birds with overexpression of *NR2B* outside of LMAN or with the green fluorescent protein (GFP) in LMAN. Our results suggest that low *NR2B* subunit expression in adult LMAN is important in conserving features of stereotyped adult courtship song.

## Introduction

Similar to speech in humans, song learning in vocal learning birds occurs most predominantly within a critical period during development^1–3^. Among avian species, zebra finches are “closed-ended” vocal learners and display little ability to further modify their vocalizations beyond the critical period as adults, when their songs become highly stereotyped^1, 4–7^. However, studies show that even in a closed-ended vocal learner, there are dynamic changes in sensorimotor processes where song stereotypy and the resistance to modify songs increases with age^8, 9^.Therefore, production of stereotyped songs in adult male zebra finches represents an attractive model system to test hypotheses on neural and genetic mechanisms underlying song stereotypy^10^.

**Figure 1:**
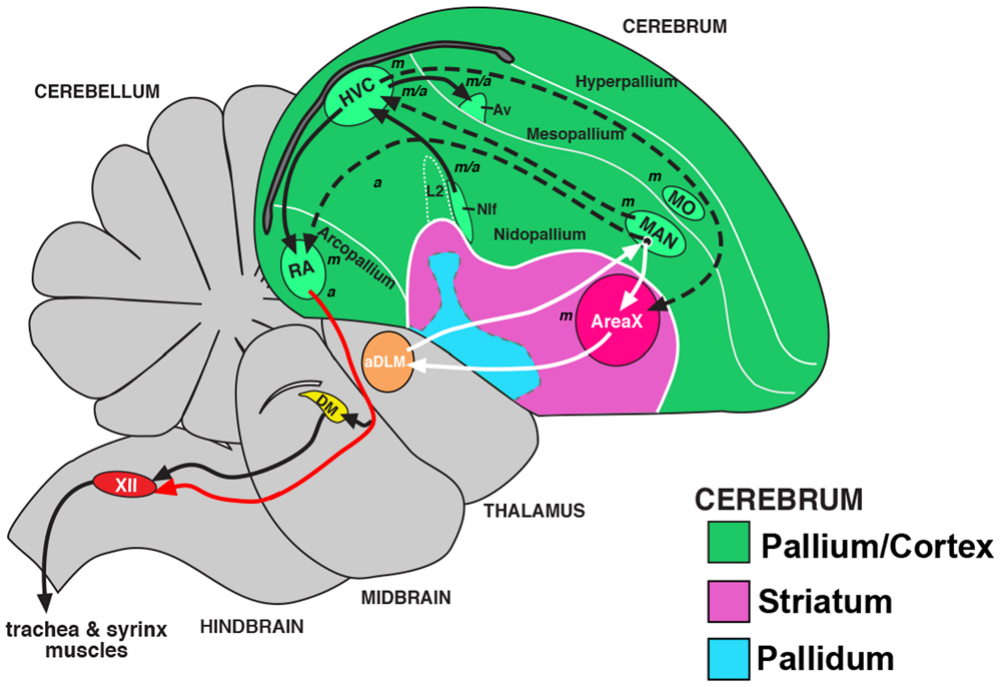
Schematic of brain pathways controlling song in songbirds. Figure modified from Chakraborty and Jarvis^23^. Black arrows, posterior vocal motor pathway; White arrows, anterior vocal learning pathway; Dashed arrows, connections between the two pathways; Red arrow, specialized direct projection from forebrain to brainstem vocal motor neurons in vocal learners. Italicized letters indicates that these regions mainly show motor *(m)*, auditory *(a)*, equally both motor and auditory *(m/a)* neural activity or activity-dependent gene expression in awake animals. Not all connections are shown, for simplicity. Abbreviations: Av, avalanche; aDLM, anterior dorso-lateral nucleus of the thalamus; DM, dorsal medial nucleus of the midbrain; H, hyperpallium; HVC, a vocal nucleus (no abbreviation); L2, field L2; M, mesopallium; MAN, magnocellular nucleus of the anterior nidopallium; MO, oval nucleus of the anterior mesopallium; N, nidopallium; Nif, interfacial nucleus of the nidopallium; RA, robust nucleus of the arcopallium; XII, 12^th^ nucleus, tracheosyringeal part.

Vocal learning requires a complex and specialized neural circuitry^11–13^. All vocal learning birds have seven forebrain song nuclei necessary for learning and producing learned vocalizations (Fig. 1)^14–17^. These forebrain song nuclei are distributed in two pathways (Fig. 1): (1) an anterior song pathway that includes a cortical-striatal-thalamic loop connecting MAN (magnocellular nucleus of the anterior nidopallium) in the cortical analog, to Area X in the striatum, to DLM (dorsal lateral nucleus of the medial thalamus) in the thalamus, and back to MAN, which is necessary for vocal learning and modifying vocalizations in different social contexts^18–21^; and (2) a posterior vocal pathway that projects from the pallial-cortical song nucleus HVC (a vocal nucleus, no abbreviation) to RA (robust nucleus of the arcopallium), to the midbrain vocal center DM (dorsal medial nucleus of the midbrain) and brainstem (nXIIts) vocal motor neurons that control the muscles of the syrinx, necessary for producing learned vocalizations. The song nuclei are adjacent to non-vocal movement activated regions^22^, and are proposed to have arisen by duplication of these preexisting motor areas^23^, suggesting that discoveries in the song learning systems could be relevant to understanding sensorimotor learning broadly.

The lateral part of MAN (LMAN) is a critical output node of the vocal cortical-basal ganglia-thalamic circuit onto RA. Although LMAN is not necessary for singing, neurons in LMAN show strong singing-related immediate early gene expression^20^ and neural activity that is correlated with song structure^24, 25^. Artificial manipulation of LMAN by microstimulation while the bird sings induces real time changes in spectral features (e.g. fundamental frequency and amplitude) of individual song syllables^4^. The dependence on LMAN for song modification is stronger during the juvenile critical period of song learning^26–28^. However, LMAN lesions in adult male zebra finches also prevent song degradation caused by deafening^27, 29^. Furthermore, inactivation of LMAN reduces some song acoustic variability seen in adult songs, and also in juvenile songs that are engaged in sensorimotor song learning from tutors, indicating that behavioral variability actively requires LMAN and cortical-striatal-thalamic integration^5, 21, 30, 31^. A similar role has been proposed for Broca’s area in the anterior cortex and the connected striatal regions in human speech, based on lesions, physiology/imaging, connectivity, and gene expression^11, 32^ (but see Long *et al*.^33^ for an alternative interpretation of HVC playing a similar role as Broca’s area based on cooling experiments, instead of HVC being similar to the laryngeal motor cortex as proposed in refs 11 and 32).

These developmental changes in LMAN are correlated with developmental changes in the *NMDAR* (N-methyl-D-aspartate receptor) subunits involved in neural plasticity, *NR2A* (N-methyl-D-aspartate receptor 2A) and *NR2B* (N-methyl-D-aspartate receptor 2B)^34, 35^. During the critical period for song development, *NR2B* mRNA levels in LMAN are higher than *NR2A* ^34, 35^. Then as the song becomes crystallized, *NR2B* expression and currents decreases, and *NR2A* increases in LMAN^34–36^, causing *NMDAR*-mediated synaptic currents to shorten (presumably with NR2B or NR2A dimerizing with the ubiquitous NR1 subunit)^37^. By the time the animals become adults, *NR2B* mRNA is lower, whereas *NR2A* mRNA is higher in LMAN compared to the surrounding nidopallium region (adult patterns also seen in the two other vocal learning lineages, parrots and hummingbirds)^38^. Importantly, *NDMARs* are necessary for normal song development^35, 39^, and LMAN input to RA is regulated primarily by *NMDAR* ^40–42^. These findings are consistent with the hypothesis that the *NR2B* gene confers increased plasticity to neural circuits^43–45^, and that its downregulation in adult LMAN may contribute to the molecular mechanisms of increased song stereotypy in adults.

Here, we tested this hypothesis by performing lentivirus-mediated overexpression of the human *NR2B* in LMAN. We found that this manipulation induced a number of changes to song, including stuttering, slower song tempo, reduced song matches to their original song, higher acoustic pitch, and altered song sequencing, approaching some characteristics found in juvenile song.

## Results

**Figure 2:**
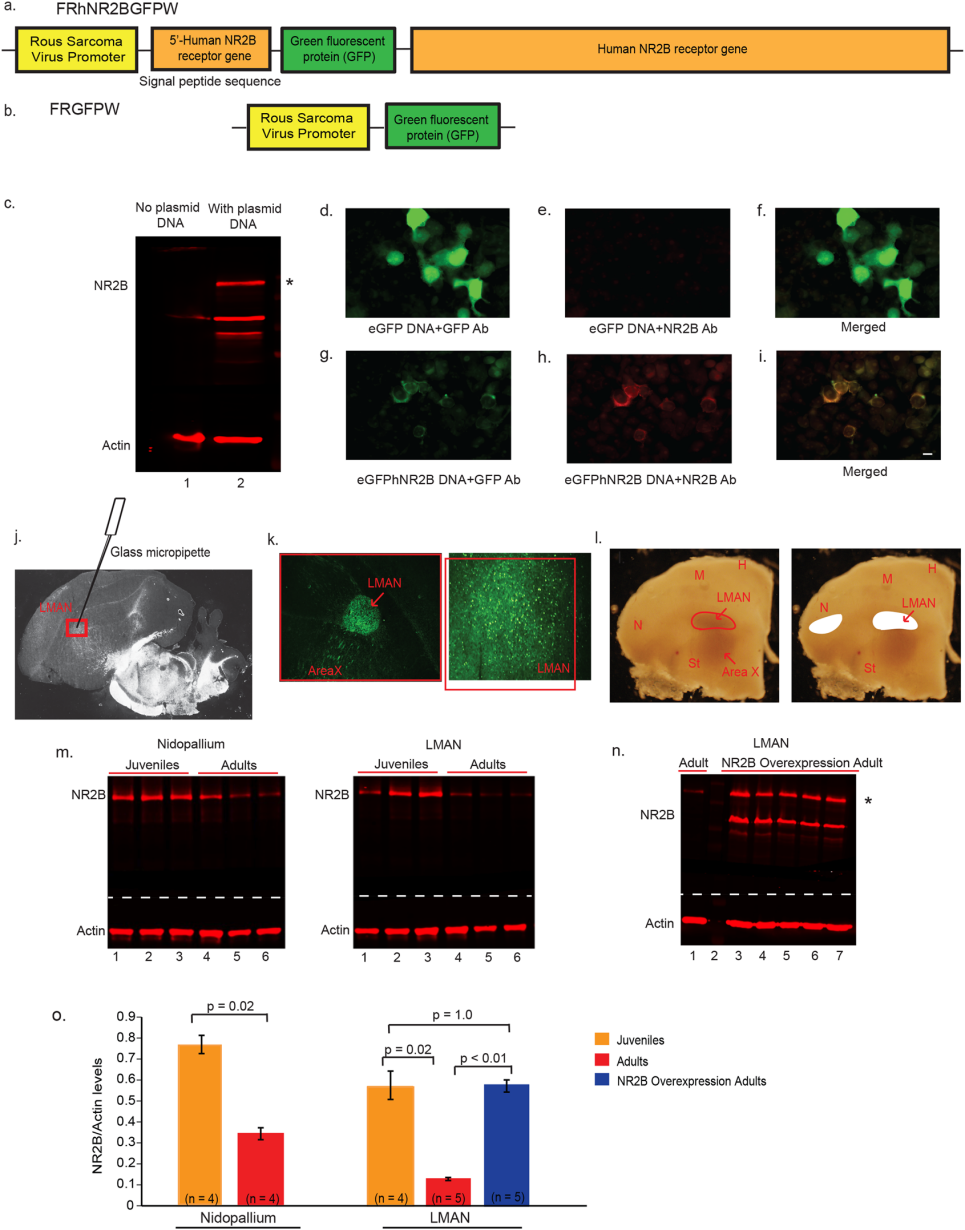
Design of lentiviral vectors for *NR2B* manipulation in LMAN. (**a**) Diagram of the eGFPhNR2B vector to overexpress human *NR2B*. (**b**) Diagram of the eGFP vector for control injections. (**c**) Western blots showing no human NR2B protein in HEK293FT cells without transfection (Lane 1), and greater NR2B protein (* ~180 kD band) in cells transfected with the eGFPhNR2B plasmid (possible degraded or unfinished NR2B protein product is detected as an additional lower band) (Lane 2). (**d**) HEK293FT cells transfected with the eGFP plasmid show GFP-expressing cells labeled using a GFP antibody (green). (**e**) Cells transfected with the eGFP plasmid stained with an anti-NR2B antibody show no labeled cells. (**f**) Merged image of (**d**) and (**e**). (**g**) Cells transfected with the eGFPhNR2B plasmid show GFP-expressing cells (localized in cell membranes) labeled using a GFP antibody (green). (**h**) Cells transfected with the eGFPhNR2B plasmid show NR2B-expressing cells (localized in cell membranes) stained with anti-NR2B antibody (red). (**i**) Merged image of (**g**) and (**h**) showing complete overlap of the human NR2B tagged with eGFP (yellow). (**j**) Darkfield view of a brain section showing the injection site in LMAN. (**k**) Cells expressing GFP-tagged (green) human NR2B ~3 weeks after injection of the eGFPhNR2B lentivirus in LMAN. (**l**) Darkfield photograph of coronal sections showing drawings of LMAN and part of nidopallium that was microdissected for protein analysis. (**m**) Western blots showing NR2B protein and actin protein from microdissected tissues of nidopallium and LMAN in juveniles and adult animals (3 animals shown as examples). (**n**) Western blot comparing endogenous NR2B protein and actin in LMAN of an adult bird (Lane 1) with additional NR2B overexpression in birds with eGFPhNR2B lentivirus in LMAN ~35–40 days after surgery (Lanes 3–7; * ~180 kD NR2B protein). Protein Standard is included in Lane 2. (**o**) Quantification of NR2B protein levels normalized to actin levels in Western blots. Even though the values were higher in all 4 animals, protein levels were not significantly higher in nidopallium relative to LMAN in juveniles (p = 0.125) possibly due to low sample size, but were higher in nidopallium compared to LMAN in adults (p = 0.05). Band density was quantified using ImageJ and results analyzed using two sample Wilcoxon tests for comparisons among groups, or paired Wilcoxon signed-rank tests for within-group comparisons. Scale bars, 10 μm (**d–i**).

To test the hypothesis that *NR2B* levels in adult LMAN influence song stereotypy, we developed a lentiviral construct eGFPhNR2B (Fig. 2a) containing the human *NR2B* tagged with enhanced GFP (green fluorescent protein), overexpressed it in LMAN of male zebra finches (NR2B group), and examined singing behavior. Our lentiviral vector was designed off of a previous construct that was shown to have all the properties of *NR2B in vivo* and *in vitro* in mammalian neurons^46^. We decided to test the human gene, because the zebra finch homolog was not completely assembled in the zebra finch genome^47^, and the *NR2B* subunit gene is highly conserved, sharing more than 90% amino acid sequence identity between the human and zebra finch^38^. We generated two control groups: (1) Birds injected with the eGFPhNR2B lentivirus in the motor nidopallium area laterally adjacent to LMAN; and (2) a lentiviral construct expressing eGFP without human *NR2B* (Fig. 2b) injected into LMAN. When we found no statistical differences between these two control groups, we grouped the data together as one large control group. We first describe the results of the validation of NR2B protein expression from the lentiviral construct, followed by the behavioral results.

### Rous Sarcoma Virus (RSV) promoter drives expression of human *NR2B* in HEK293FT cells

To determine if the custom designed eGFPhNR2B lentiviral plasmid expressed the eGFP-tagged human NR2B protein, we transfected the plasmid in human embryonic kidney 293FT cells (HEK293FT), which normally do not express endogenous NR2B protein^48, 49^. In such constructs, the amino signal transport sequence upstream of the GFP molecule has been shown to be cleaved off *in vivo* to successfully transport a fused GFP-NR2B functional protein into the cell membrane^46^. Consistent with these past constructs, compared to control cells without transfection (Fig. 2c, Lane 1), cells transfected with our eGFPhNR2B lentiviral plasmid expressed high levels of the human NR2B protein of the expected ~180 kD size, as measured by Western blots using a NR2B antibody (Fig. 2c, Lane 2). As measured by immunocytochemistry, cells transfected with the eGFP only and eGFPhNR2B plasmids showed GFP labeling (Fig. 2d,g), and as expected only the eGFPhNR2B transfected cells showed NR2B labeling (Fig. 2e,h). The GFP labeling in both the eGFP only and eGFPnNR2B transfected cells was localized to the cell membrane (Fig. 2h), and for the latter construct, the eGFP label was distinctly 100% co-localized with NR2B labeling in the membrane (Fig. 2g–i). These findings indicate that our eGFP-tagged NR2B human protein is successfully expressed in cells *in vitro* by the RSV promoter, is in the correct frame in order to be recognized by the NR2B antibody, and is transported to the membrane as expected.

### RSV promoter drives expression of human *NR2B* gene in LMAN in the avian brain

To determine if these constructs could infect LMAN cells and overexpress the human NR2B protein *in vivo* in the avian brain, we performed bilateral injections of the eGFPhNR2B, or eGFP lentiviruses in LMAN of adult male zebra finches (Fig. 2j; n = 4 each). Three weeks after injections, we analyzed serial brain sections by immunocytochemistry and found strong GFP-labeled cells in most of the LMAN song nucleus with both constructs (see Fig. 2k for example). As found *in vitro*, *in vivo* expression was localized to the cell membrane.

To quantify the amount of NR2B overexpression and determine if the known developmental changes in mRNA expression^35^ are translated to protein expression, we measured NR2B protein levels by Western blots on microdissected LMAN song nuclei and a comparable sized region of the adjacent lateral nidopallium of juvenile and adult birds (Fig. 2l) following a protocol used previously in our laboratory^50^. We calculated the normalized NR2B protein levels to the actin levels of each sample. In all animals without viral injections, we detected the endogenous ~180 kD NR2B protein (Fig. 2m). Relative to 30-day old juveniles, NR2B showed decreased protein expression in both LMAN and the adjacent nidopallium in adults (Fig. 2m,o). Also in both juveniles and adults, expression in LMAN was lower than the adjacent nidopallium, but the developmental decrease in LMAN was larger (Fig. 2m,o), consistent with what is seen anatomically at the mRNA level using *in situ* hybridizations^34, 35^. Importantly, injections of the eGFPhNR2B lentivirus in LMAN in adults brought back NR2B protein levels to the same as those seen in juveniles (Fig. 2n,o). Because the predicted size of the GFP-tagged NR2B protein is only 6 kD larger than the endogenous NR2B protein, the ~180 kD band that we detected likely reflects both endogenous and overexpressed human NR2B. In the transfected brain regions (Fig. 2n), as well as in the transfected cultured cells (Fig. 2c), there is an additional lower molecular weight band, which we interpret to be possibly a processed alternative isoform of NR2B generated from our vector, or a degraded or unfinished protein product of NR2B. There was no detectable change in actin expression levels in the control and injected LMAN samples, indicating that the increased NR2B levels detected is not due to a general increase in protein expression or overloading of the Western blots for the experimental condition.

**Figure 3:**
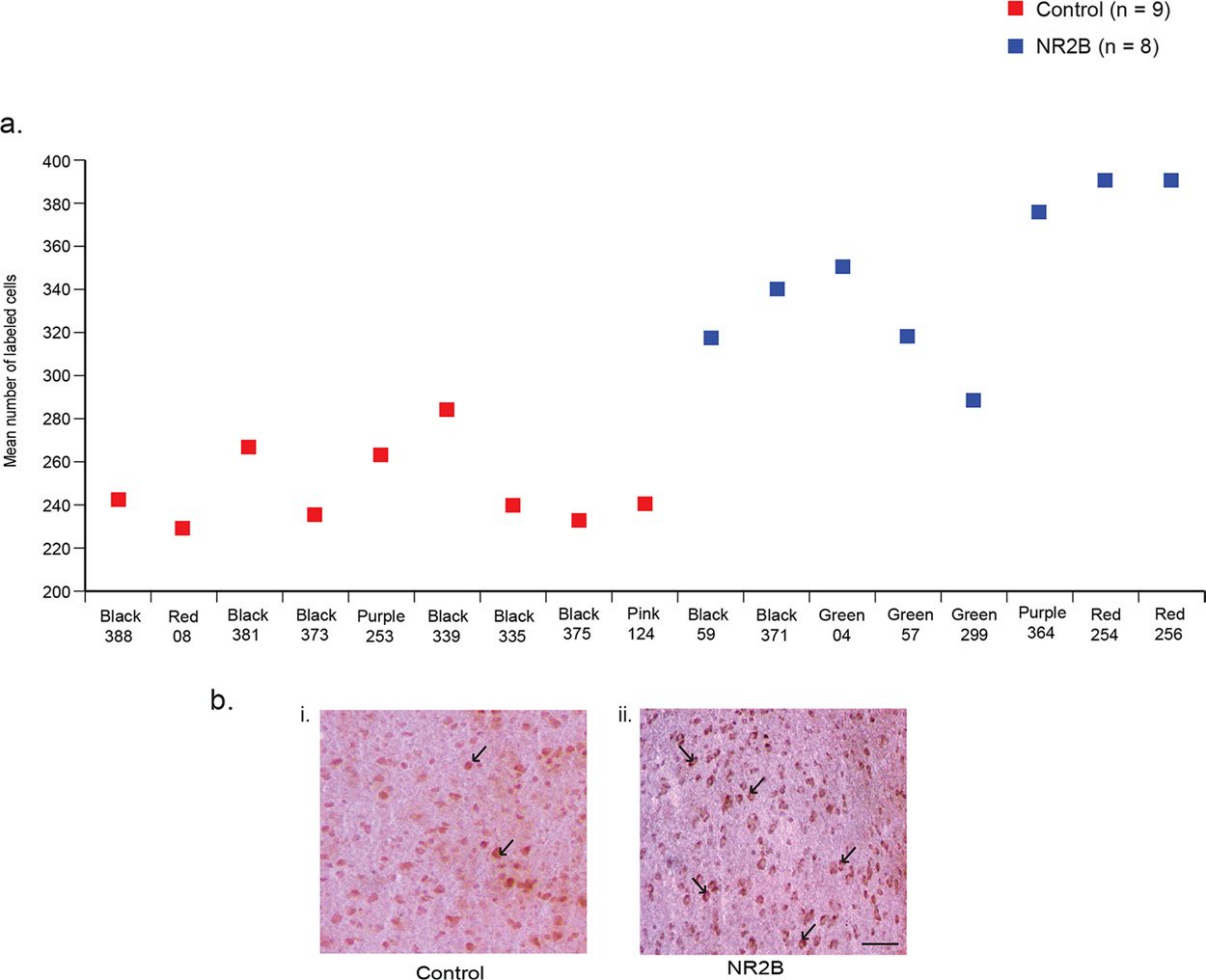
Immunostaining of NR2B protein in LMAN cells. (**a**) Quantification showing higher levels of labeled cells in birds injected with the eGFPhNR2B lentivirus in LMAN (blue) compared to control birds with injections of the eGFPhNR2B lentivirus in nidopallium or the eGFP lentivirus in LMAN (red). Control-injected animals did not differ in number of labeled cells (p = 0.73, two sample Wilcoxon test). (**b**) Examples of labeled LMAN cells in eGFP-injected animals (i), and in eGFPhNR2B-injected animals (ii). Scale bar, 200 μm.

We performed immunohistochemistry using the NR2B antibody on frozen serial brain sections of animals that were injected and recorded for song behavior for up to 2.5 months. Animals injected with the eGFPhNR2B lentivirus in LMAN had significantly more cells expressing high levels of NR2B protein compared to the control animals (Fig. 3a,b; expression level determined by unbiased threshold function and cell counts made blind to group; see methods). These results also show that the overexpression of NR2B protein in the brain was long lasting.

### Overexpression of human *NR2B* in LMAN results in stuttering, and increases in song tempo and song sequence variability

#### Stuttering

**Figure 4:**
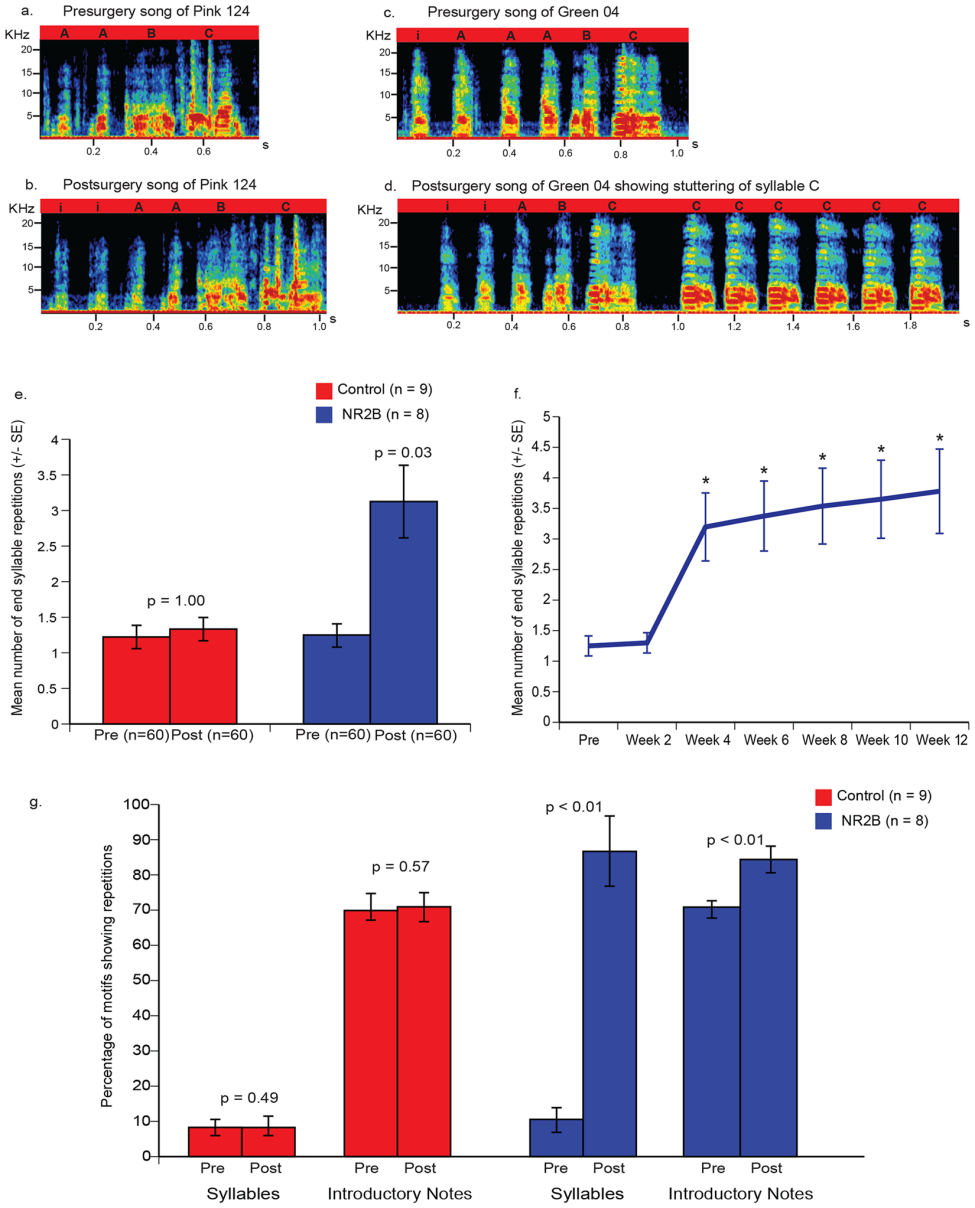
Representative sonograms of songs and mean end syllable repetitions before (presurgery) and after (postsurgery) injections. (**a**) Presurgery song of a control bird Pink 124. (**b**) Postsurgery song of Pink 124 injected with eGFPhNR2B lentivirus in the motor nidopallium showing no change in song after injections. (**c**) Presurgery song motif of a bird Green 04. (**d**) Postsurgery song motif of Green 04 showing end motif syllable stuttering after overexpression of human NR2B in LMAN. Black letters indicate syllable types in song motifs. Sonograms were generated using Avisoft-SASLab Pro software (Avisoft Bioacoustics, Germany). (**e**) Mean number of end syllable repetitions in control and NR2B groups of birds. (**f**) Time course of mean end syllable stuttering in birds (n = 8) of the NR2B overexpression group that were injected with the eGFPhNR2B lentivirus in LMAN. Pre indicates end syllable repetitions before injections. Each time point is an average of 10 song bouts; weeks 2–12 were compared to presurgery levels using paired t tests; asterisk represents p value < 0.01. (**g**) Percentage of motifs (mean ± SE) showing end syllable and introductory note repetitions in birds of the controls and NR2B groups. We used a cut-off of more than 3 repeats of introductory notes at the beginning of each motif of a song bout to be included as repetitions in our analyses for quantification. P values were determined using paired Wilcoxon signed-rank tests.

**Figure 5:**
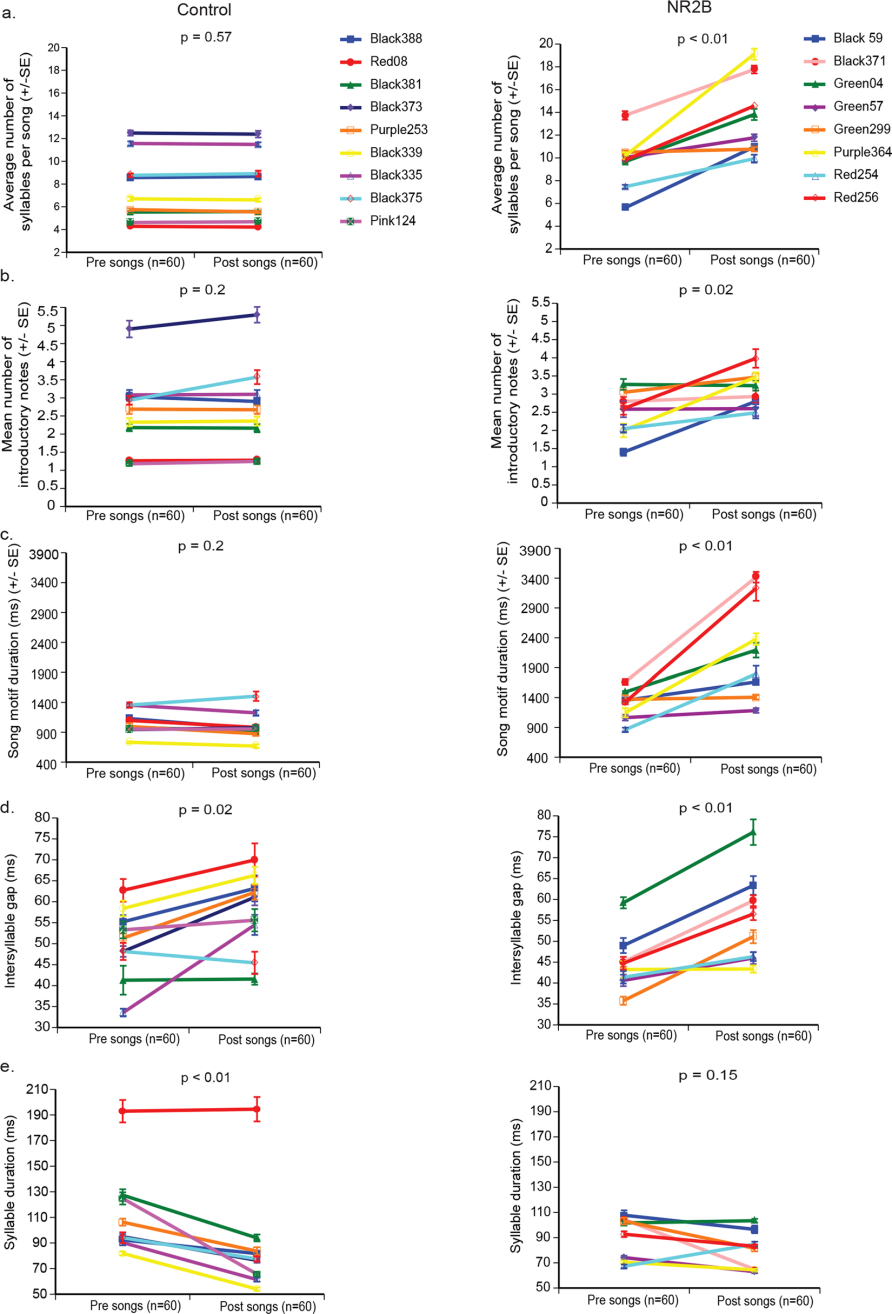
Overexpression of human NR2B induced changes in song motif structure. (**a**) Average number of syllables per song motif. (**b**) Mean number of introductory notes per song motif. (**c**) Song motif duration. (**d**) Intersyllable gap durations. (**e**) Average of all syllable durations (bird Red08 of the control group appeared to be an outlier, which was confirmed by a Dixon’s test in R in both presurgery (p = 0.02) and postsurgery (p < 0.0001) conditions, and thus not included in the statistical analyses of the entire group. Each line is one animal presurgery and postsurgery, mean ± SE, with p values determined using paired Wilcoxon signed-rank tests.

To assess the potential impact of *NR2B* overexpression in LMAN on singing behavior, we first focused on global structure of the song motifs. The most auditorily and visually apparent result on sonograms, not often seen in adult or juvenile birds, was stuttering (Fig. 4a–c). Assessed quantitatively, before surgery, the mean number of syllables produced across motifs did not differ between birds selected for control (7.58 ± 0.98) and NR2B overexpression (9.62 ± 0.83) injections prior to surgery (Fig. 5a; p = 0.14; two sample Wilcoxon test). The mean number of syllables in the motifs of the control birds also did not change post-surgery (7.56 ± 0.99; Fig. 5a). In contrast, birds with NR2B overexpression in LMAN showed increased number of syllables per motif (13.58 ± 1.19; Fig. 5a). This increase was in part due to increased end syllable repetition, characteristic of stuttering (Fig. 4c–e). All the birds that repeated syllables, particularly at the end of the motifs, started to do so by 4 weeks after surgery (Fig. 4f), and continued to stutter at the time we sacrificed them 3 months after surgery to collect their brains. This timing indicates that it was the NR2B overexpression that caused the stuttering, and not possible mechanical injury to LMAN from the surgery, which would have been immediate. The total number of introductory notes at the beginning of the first motif of a song bout, which can be several per motif and more with directed singing, also increased in some of the NR2B overexpression birds (Fig. 5b). The percentage of motifs showing stuttering was high, which went from an average of ~10% and ~72% for end syllable and introductory notes respectively, to ~87% and ~85% after NR2B overexpression (Fig. 4g). No such changes were seen in control birds (Fig. 4g, controls combined). Since stuttering is not normally found in juvenile birds, these findings indicate that NR2B overexpression in adults result in aberrant stuttering.

#### Song tempo

Because there is a dramatic increase in song tempo (produced faster) from juvenile development to adulthood^51^, and because song tempo is also known to change throughout the day^52^, we measured the songs consistently at the same time of day for all animals throughout the course of the recordings. Whereas control birds (both groups combined) showed no change before and after surgery, the NR2B LMAN overexpression group showed an average 1.7-fold increase in song motif duration (Fig. 5c). This increase was due more to the increased number of syllables (i.e. stuttering), as we found that the mean intersyllable gaps between all syllables increased in both control and NR2B LMAN overexpression birds (Fig. 5d). However, in controls, this was associated with a concomitant decrease in the total average syllable duration, which presumably compensated for the silent gap increases, resulting in no overall change in total motif duration of the control group (Fig. 5c). The NR2B group did not show this concomitant decrease in average syllable duration (Fig. 5e). This suggests a natural continued age-dependent decrease in syllable duration (independent of silent gap increases) past the critical period, which was blocked with *NR2B* overexpression. Taken together, we infer that NR2B overexpression in LMAN partially reversed and further prevented the age-dependent tempo increases in adult birds, where they become more like juvenile birds.

#### Song sequence

**Figure 6:**
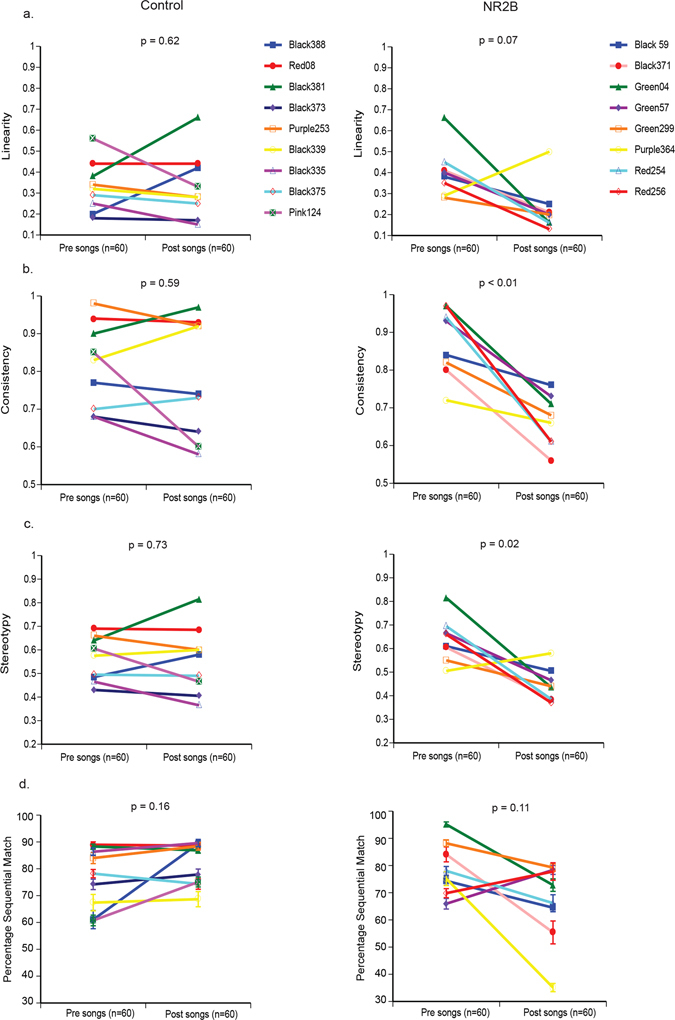
Song sequencing changes after overexpression of human NR2B in LMAN. (**a**) Song motif linearity. (**b**) Song motif consistency. (**c**) Song motif stereotypy. (**d**) Percentage sequential match. Each line is one animal presurgery and postsurgery, mean ± SE, with p values determined using paired Wilcoxon signed-rank tests.

To quantify syllable sequencing, we used commonly studied measures of sequence linearity, sequence consistency, and sequence stereotypy of zebra finch songs^29, 53, 54^. Juvenile birds are known to have higher variability in these measures compared to adults^5, 55^. In control adult birds (both groups combined), we found considerable individual variability pre and post surgery, but overall group means for all three sequence measures remained unchanged (Fig. 6a–c). In contrast, consistent with our hypothesis, the NR2B overexpression LMAN group exhibited dramatically lower group mean scores of consistency and stereotopy, and a strong trend for linearity (significant with the outlier Purple364 removed, p = 0.02, paired Wilcoxon signed rank test; Fig. 6a–c). There was however, no significant group change in percentage sequential match (Fig. 6d), a measure of how temporally similar a sequence is from one song to another. Thus, overexpression of *NR2B* reduced the characteristic features of stereotyped sequencing of adult courtship songs by changing the songs to be more variable from one rendition to another.

### Overexpression of human *NR2B* in LMAN cause increased overall song syllable acoustic similarity, changes in pitch, but not in mean acoustic features

#### Syllable acoustic similarity and accuracy

**Figure 7:**
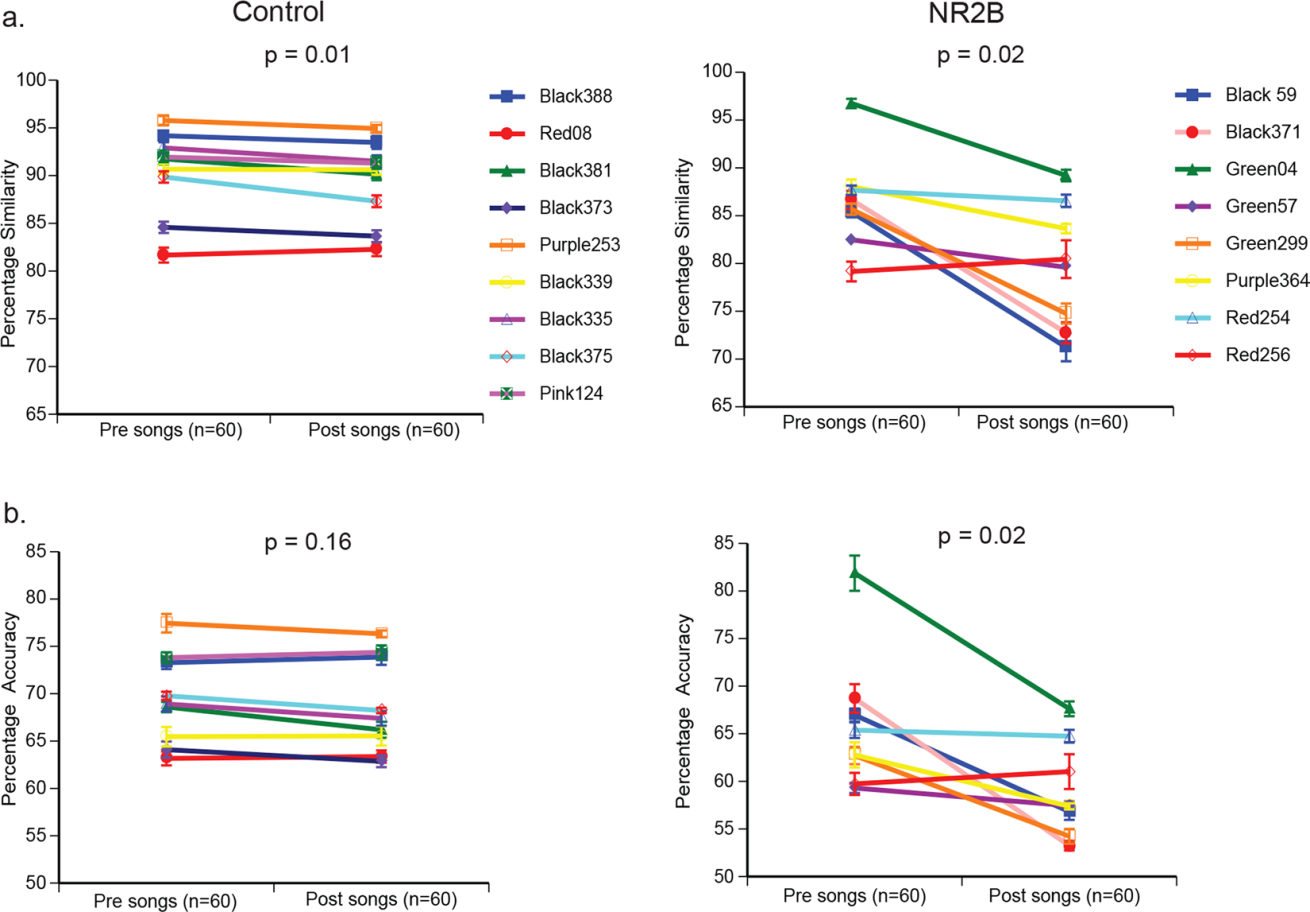
Song similarity changes after overexpression of human NR2B in LMAN. (**a**) Percentage similarity before and after injections in control or NR2B groups. (**b**) Percentage accuracy. Each line is one animal presurgery and postsurgery, mean ± SE, with p values determined using paired Wilcoxon signed-rank tests.

Because LMAN is known to control the level of variability in acoustic structure of song syllables^4, 30, 56^, and these are more variable in juveniles than adults, we measured the overall degree of song syllable acoustic similarity pre versus post-surgery (a measure that summarizes multiple acoustic features into one value; see methods). There was a very small (mean change from pre to post = 1.10 ± 0.36), but significant decrease in the control group in the degree of percentage similarity from one song motif to another song motif (Fig. 7a), indicating a slight increase in variability of acoustic features with age or due to the surgery. However, in the NR2B LMAN overexpression group, for most birds there were bigger decreases (mean change from pre to post = 5.6 ± 1.92) in overall similarity (Fig. 7a). Consistent with these findings, the NR2B overexpression group, but not controls, showed a robust and significant decrease in percentage accuracy (Fig. 7b).

#### Syllable structure

We next measured which specific syllable acoustic features changed, including pitch, fundamental frequency, entropy, goodness of pitch, and amplitude modulation, all of which are known to be sensitive to LMAN perturbations^27, 29, 30, 54^ and some of which are more variable in juvenile birds. We found increased mean pitch in the NR2B group (Supplementary Fig. S1a), but no significant group mean differences of the other four acoustic parameters (Supplementary Fig. S1b–e). To examine variability more directly, we calculated the standard deviation (SD) of each of the acoustic features, but found no significant differences in overall SD variability in any of the syllable acoustic features we analyzed (Supplementary Figs S2 and S3). For a second measure, we also did not detect any significant changes in group means for the coefficient of variation (% c.v) of the syllable acoustic features in the control or NR2B groups (Supplementary Fig. S4).

These findings indicate that except for pitch, the decreased similarity and accuracy maybe best explained by an interacting combination of acoustic features showing greater variability with *NR2B* overexpression in LMAN.

### Manipulated protein levels in LMAN correlate best with song motif duration and sequence match

**Figure 8:**
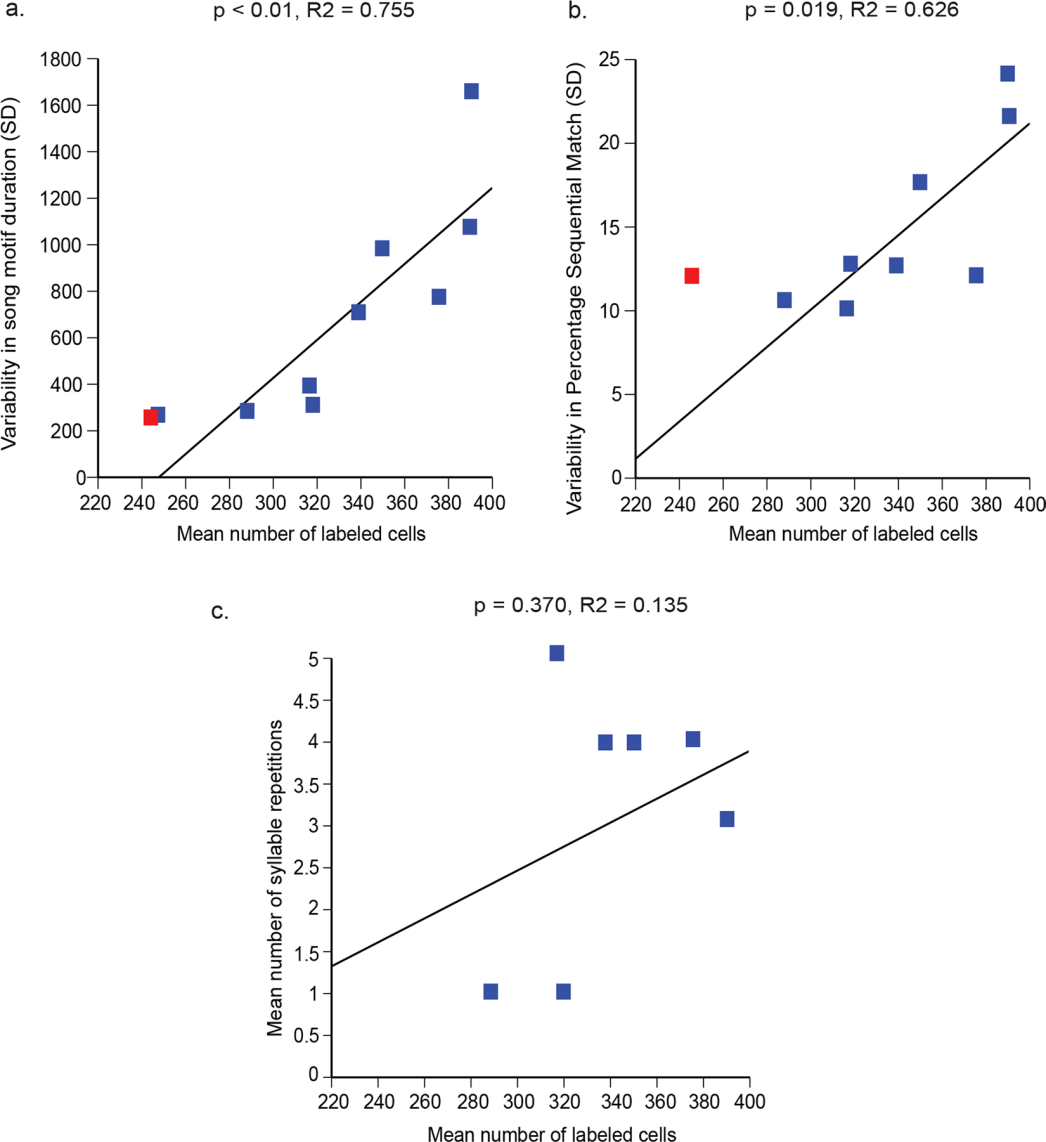
Correlations between number of cells expressing high levels of NR2B protein in LMAN and song features in animals injected with the eGFPhNR2B lentivirus in LMAN. (**a**) Protein levels of NR2B in LMAN are correlated with variability in song motif duration after injections. (**b**) Protein levels of NR2B in LMAN are also correlated with variability in percentage sequential match after injections. P and R^2^ values were determined by regression analyses. Red boxes represent the control groups mean variability in song motif duration (**a**), and percentage sequential match (**b**) that were not included in the regression analyses and are shown for comparison only. (**c**) Protein levels of NR2B in LMAN are not correlated with mean number of syllable repetitions.

Here, we explicitly addressed the issue of causality to determine whether animals with manipulated overexpression of NR2B protein in LMAN have differences in song sequencing and acoustic features with the proportion of cells expressing NR2B. This would also allow us to determine more subtle changes that are sensitive to NR2B expression levels. We found a strong correlation between mean number of NR2B-labeled cells (high protein levels) with variability in two sequence measures: variability in mean song motif duration (Fig. 8a) and variability in percentage sequential match (Fig. 8b). Although, we did not include the control groups in our regression analyses, the control group means (red boxes in Fig. 8) were at the lower end of the range for the NR2B group (blue boxes in Fig. 8), consistent with the levels or number of cells expressing NR2B influencing these song features. ﻿Interestingly, we did not detect any significant correlations with mean number of syllable repetitions and the number of NR2B-expressing cells ﻿(Fig. 8c). We also did not detect significant correlations with other song sequencing or acoustic features (Supplementary Figs S5 and S6). Overall, our results indicate that NR2B has an important role in modulating variability in the temporal, sequencing, and some acoustic features of songs.

## Discussion

Our study provides important advances on understanding the role of the *NR2B* subunit gene in production and maintenance of stereotyped courtship song in adult zebra finches. Here we tested the hypothesis that the developmentally down-regulated levels of *NR2B* may be important in conferring song stereotypy in adult males. We discovered that overexpression of *NR2B* bilaterally in LMAN causes aberrant repetition of beginning (introductory notes) and end-syllables similar to stuttering, and reversion back to more of a juvenile state to the lengthening of the song motif duration and increased variability of song sequencing and global acoustic structure. Considering that in adult zebra finches lesions of LMAN do not result in robust changes in the overall structure of the song^6, 21, 57^, or in variability in syllable sequencing^6, 21, 30^, these findings indicate that overexpression of *NR2B* to a more juvenile state reactivates a more dominant role of LMAN in influencing song plasticity. Below we discuss the implications of our findings within the context of utilizing zebra finches as a model to explore cortical-basal-ganglia circuit function and dysfunction.

The syllable repetition findings have important parallels to human dysfluencies, namely stuttering and verbal apraxia. The increased syllable repetitions we observed shares many features of developmental and neurogenic stuttering observed in humans who have suffered damage to the cortical-basal ganglia circuit^58^ or increased activation in this circuit; the more a person stutters, the more neurons of this network are activated^59^. Developmental stuttering refers to speech repetitions or prolongations that affect mostly children at a young age, whereas the rare neurogenic stuttering or acquired stuttering is due to adult brain lesions and have been described to be a core aspect of some aphasias^60–62^. In humans, individuals with neurogenic stuttering are more predisposed to stutter at the beginning of a sentence^63, 64^, although other dysfluencies have been reported where stuttering occurs at the end of the final sounds^65, 66^. The repetitions at the end of the motifs we observed usually occurred after the first motif of a bout, suggesting that the birds become stuck in sequencing from one motif to the next. In humans, motor-speech disorders such as verbal apraxia are often associated with repetitions of phonemes similar to stuttering^67, 68^. Theories posit that an important mechanism causal to stuttering is impairment of the basal ganglia circuits that produce timing cues for the initiation of motor movement in speech^58^. Our findings in zebra finches support this view.

We do not believe that this induced stuttering from *NR2B* overexpression in LMAN is a reversion to the juvenile state. Only a small percentage of a population (up to ~7%) of laboratory-raised male zebra finches are naturally known to stutter^69^. Although stuttering can be learned from adult tutors in juvenile zebra finches^69^, it is not known to be part of the normal song learning process. Rather, we believe that this is aberrant behavior due to *NR2B* overexpression in adult LMAN. Previous studies have reported that adult LMAN lesions do not affect introductory notes^30^ nor cause stuttering for other syllables^54^. Kubikova *et al*.^54^ found a high incidence of stuttering at the end of the song motifs with Area X neurotoxic lesions, but only in birds that already had a predisposition for a small number of repeats before the lesions^54^. We suggest that while lesion inactivation of LMAN does not induce stuttering, activation or enhancement of its function through *NR2B* overexpression causes the birds to have difficulty sequencing between motifs, by getting stuck at the beginning and end syllables when switching from one motif to another. Syllables produced in between or at the end of motifs are more variable and less stereotyped than those within the motif^28^, possibly making them more sensitive to perturbations.

Consistent with the stuttering findings, our findings of increased song motif tempo in the *NR2B* overexpression (i.e. LMAN activation) group is the opposite to that found with lesion inactivation of LMAN in zebra finches^27, 54^, indicating the change we observed is a gain in LMAN function with *NR2B* overexpression. The normal tempo changes of zebra finch song over juvenile to adult development, could be due to stretching and compressing proportionally syllables and silent gaps^52^. Consistent with our findings in control birds, gaps, but not syllables are known to shorten over the first year of life of an adult bird^51^, and recently it has been shown to be more genetically controlled than learning syllable imitation in zebra finches^70^. Other studies have reported no decreases in intersyllable gaps, but a decline in syllable durations over 1.5 years of the adult life of a male^71^, whereas studies in Bengalese finches report that songs slow down later as the bird ages, primarily due to increase in intersyllable gaps^72^. It is unclear why the findings from these studies are equivocal, but they may be attributed to methodological differences. We speculate that *NR2B* in LMAN is likely important in modulating the variability in syllable duration, and not intersyllable gaps, since gaps appeared to increase in both control and NR2B groups. We also speculate that the higher levels of *NR2B* in manipulated adults induced a partial juvenile-like state, by preventing syllable durations to shorten over time, while simultaneously allowing the intersyllable gaps to increase.

We were surprised to find that *NR2B* overexpression in LMAN had a larger impact on song sequencing relative to syllable acoustic features, since variability in such syllable acoustic features is reduced after LMAN lesions before they impact sequencing, consistent with the idea that its target nucleus RA is involved more in controlling syllable acoustic structure than sequencing^73, 74^. Instead, our results are consistent with those reported for Area X lesions^54^, where the authors noted more apparent sequencing changes in adult animals. This is perhaps due to our manipulation enhancing LMAN function with an intact Area X. Nevertheless, we still found some syllable acoustic changes, such as decreases in stereotypy and increases in pitch after *NR2B* overexpression, indicating that *NR2B* levels in LMAN impact acoustic features to some extent. In adult zebra finches, microstimulation of LMAN induces variability in pitch^4^, whereas lesions or ablation of LMAN results in prolonged reduction in the variability of syllable pitch^29, 30, 75, 76^. We speculate that *NR2B* function in LMAN may have a more dominant role in controlling sequencing behavior either through intrinsic actions or its action on the downstream nucleus AreaX, and have a less dominant role in acoustic behavior through its action on RA.

To explain our findings at a molecular level, we propose the following hypothesis: Since *NR2B* relative to *NR2A* causes the NMDA receptor channel complex with NR1 to stay open longer^44, 77^, increased *NR2B* expression could slow down songs by increasing channel opening time, preventing the syllables from compressing in length as the bird ages, among other effects. The increased channel opening time with NR2B has been proposed to allow LMAN to integrate multiple synaptic inputs during juvenile development for vocal learning^78^; however, song learning does not require slow NMDA-EPSCs^79^, even though blocking NMDA receptor activity during tutoring decreases the number of syllables that are learned by the juveniles^80^. In light of our findings, we hypothesize that the naturally decreasing *NR2B* during development may be also critical in ensuring the characteristic faster song tempo of courtship songs that females prefer over the slower and more variable undirected songs^18, 81, 82^.

An alternative hypothesis is that introducing the exogenous human *NR2B* in LMAN may disrupt or alter the function of the endogenous *NR2B* receptor or the overexpressed *NR2B* may not be expressed in the appropriate cell type and cause a change as a result. Since we are unaware of any other studies in songbirds that have manipulated *NR2B* or its interacting receptor levels in LMAN, we can only speculate on such a possibility from similar studies of genetic enhancement in mammals. Previous studies in mammals that have overexpressed *NR2B* in the forebrain, using a similar construct as ours, have reported enhancement of prefrontal cortical long-term potentiation (LTP) and working memory, which was abolished by blocking with an *NR2B* subunit antagonist^83^. *NR2B* subunit overexpression also enhances NMDA receptor activation and channel opening, and learning and memory in transgenic mice^84^. *NR2B* endogenous expression in our experiments also appeared in the larger cells (i.e. projection neurons) of LMAN that normally express *NR2B*. If *NR2B* were overexpressed in other cells that do not normally express *NR2B*, the protein would not be expected to function or compete with endogenous function since there would not be an NR1 subunit to bind with and no endogenous *NR2B* to compete with. Further studies will be required to adequately address the effects of *NR2B* overexpression in songbirds and other models to better understand how it affects endogenous *NR2B* function. Nevertheless, the combined findings and studies indicate that overexpression of *NR2B* in songbird LMAN and other brain regions in mammals is able to at least partially recapitulate some plastic states.

In conclusion, our findings suggest that changing the expression of one gene in adult LMAN to mimic juvenile levels is sufficient to recapitulate a partial juvenile song behavior state. This includes decreased song tempo, and increased sequence and overall acoustic variability. These findings support the hypothesis that low *NR2B* levels are important in allowing stereotypy of adult courtship songs in male zebra finches. These changes were also accompanied by an unintended side effect of stuttering, which could be also potentially explained as a sequencing change. This neurogenic stuttering in zebra finches shares some common features to human stuttering, and therefore the zebra finch model may represent a potential tractable and translational model to test hypotheses on cortical-basal ganglia circuitry dysfunctions that could lead to new therapeutic insights.

## Methods

### Lentiviral vector constructs and production

Since the zebra finch *NR2B* gene sequence was not completely assembled from the zebra finch genome^47^, we designed a custom lentiviral construct with the full-length sequence of the human *NR2B* cDNA (Open Biosystem clone ID 8322672; GenBank Accession #BC113620.1). We followed cloning techniques described in Luo *et al*.^46^ to optimize overexpression of the human *NR2B* gene, in this case in zebra finches, with an enhanced GFP protein tagged as a reporter gene driven by the RSV promoter (eGFPhNR2B; Fig. 2a). This construct design was made based off of the pEGFP-NR2B plasmid containing the rat NR2B sequence that is commercially available from Addgene (Addgene plasmid #17925). The signal peptide of the human NR2B differs from the rat (or mouse) NR2B by only two amino acids, where the arginine (R) in human is replaced with a serine (S) in rat where serine is the 5^th^ amino acid located past the signal sequence (Fig. 2a). We constructed the expression vector for eGFPhNR2B by inserting the eGFP cDNA sequence in frame with the NR2B subunit between the 5^th^ and 6^th^ codons after the predicted sequence for signal peptide where the eGFP is in place of the serine. This signal peptide is cleaved off leaving a fused amino terminal GFP and carboxy terminal NR2B protein^46^. Using a variety of experiments including electrophysiology, Luo *et al*.^46^ had reported that a similarly GFP tagged NR2B protein does not interfere with the *NR2B* protein function. Similar designs of other NMDA receptors tagged with eGFP have been published before where such constructs have been shown to be functional without any defects of channel function due to the insertion of the eGFP after the predicted signal peptide recognition sequence^85, 86^. Following previous studies^87, 88^, we also generated a self-inactivating eGFP lentivirus^89^ that contained the enhanced GFP protein alone as the reporter gene driven by the same RSV promoter (Fig. 2b).

We prepared the lentiviral vectors by transfecting 6 × 10^6^ HEK293FT cells with 5 μg of the vesicular stomatitis virus glycoprotein (VSVg) envelope encoding plasmid, 15 μg of the delta-8.9 packaging plasmid, and 20 μg of the promoter-reporter or promoter-gene of interest plasmids using Lipofectamine 2000 (Life Technologies, Grand Island, NY). After 72 hours, we harvested the supernatant from one 10 cm culture plate, filtered them with a 0.45 μm filter, and centrifuged them at 26,000 rpm for 2 hours at 4 °C to obtain the pellet. We resuspended the pellets and serially diluted the lentivirus to transduce HEK293FT cells. After 72 hours, we counted the number of labeled HEK293FT cells to calculate the viral titers (eGFPhNR2B used for brain injections was 9.9 × 10^6^ ffu/ml and eGFP was 5.2 × 10^6^ ffu/ml).

### Transfection of lentiviral constructs into HEK293FT cells

To test if the eGFPhNR2B lentiviral plasmid expressed the eGFP-tagged NR2B protein, we initially performed cell culture experiments on HEK293FT cells. HEK293FT cells were cultured in 10 cm plates in serum plus DMEM (Life Technologies) medium (10% FBS+ L-Glutamine, Pen-Strep). Thereafter, the cultures were split into 7.5 × 10^5^ cell samples plated on 6 well plates. Cells were allowed to reach 80–90% confluency and 1 μg of the eGFPhNR2B plasmid DNA was added along with 2 μl of Lipofectamine 2000 in Opti-MEM Reduced Serum medium (Life Technologies). Control wells were not transfected with any plasmid DNA. HEK293FT cells were incubated for 2 days at 37 °C after which we detected protein expression either directly on slides by immunocytochemistry and by Western blots, as described below.

### Immunocytochemistry on HEK293FT cells transfected with the eGFPhNR2B plasmid to detect *NR2B* protein

We performed immunocytochemistry using HEK293FT cells on 24 well plates on PDL coated coverslips. For all experimental conditions, 50 μl HEK293FT cells from a confluent 10 cm dish per well were transfected with 1 μg of eGFPhNR2B plasmid DNA and 1 μl of Lipofectamine 2000. For detection of GFP expression by fluorescence, we rinsed the cells with 0.1 M PBS (phosphate buffered saline) and fixed them with 1 ml of 4% paraformaldehyde for 15 min at room temperature and then washed the cells with 1 ml of cold 0.1 M PBS (3 × 5 min). The control wells were mounted with Vectashield mounting medium with DAPI (H-1200, Vector Laboratories, Burlingame, California). For detection of GFP with an antibody, in other wells we permealized the cells with 1 ml PBST (0.1 M PBS + 0.1% Triton X) for 10 min at room temperature. We blocked background for the cells with 1 ml of PBST + 1% BSA (Bovine serum albumin) for 30 min at room temperature. The primary GFP antibody (GFP Clone 3E6 Mouse IgG_2A_ monoclonal antibody, Cat. #A-11120, Molecular Probes, Eugene, OR, USA) was added at a dilution of 1:1000, and incubated with the cells at 4 °C overnight. We washed the cells (3 × 10 min) with 1 ml of 0.1 M PBS and incubated the cells with the secondary antibody (Alexa Fluor 484 Goat anti-mouse IgG, Cat. #A-11001, Molecular Probes) at a dilution of 1:1000 for 30 min at room temperature in the dark. Finally, we washed the cells in 1 ml of 0.1 M PBS (3 × 10 min) and mounted them with the Vectashield mounting medium with DAPI.

For detection of *NR2B* expression, we rinsed the cells from a separate set of wells with 1 ml of 0.1 M PBS and fixed them in 1 ml of 4% paraformaldehyde for 15 min at room temperature. We washed the cells in 1 ml of cold 0.1 M PBS three times for 5 min each and stored the cells overnight at 4 °C in PBS. On the next day we blocked background signal from the cells with 500 μl of 0.1 M PBS + 1% BSA for 30 min at room temperature. We added the mouse NR2B monoclonal primary antibody (Cat. #MA1-2014, Thermo Scientific, Rockford, IL, USA) at a dilution of 1:1000 in 200 μl of 0.1 M PBS + 1% BSA and incubated the cells at room temperature for 2 hr. We washed the cells in 1 ml of 0.1 M PBS three times for 5 min and incubated the cells with the secondary antibody (Alexa Fluor 555 Goat anti-mouse IgG_1_, Molecular Probes) at a dilution of 1:1000 in 200 μl of 0.1 M PBS + 1% BSA for 1 hr at room temperature in the dark. Finally, we washed the cells in 1 ml of 0.1 M PBS three times for 5 min and mounted them with the Vectashield mounting medium with DAPI. For all methods, expression was then detected under fluorescence microscope settings using a Leica DMI 4000b microscope (Leica Microsystems, IL, USA).

### Western Blot

When conducting Western blots to detect protein levels from both microdissected brain tissue and HEK293FT cells, the samples that were transfected with the eGFPhNR2B plasmid, or without the plasmid (control cells), were collected and stored at −20 °C until further processing. We lysed the cells from HEK293FT cells or brain tissue with SDS-PAGE 2X sample buffer (a solution containing stacking buffer (10% SDS, 80% glycerol, and 1 ml of β - mercaptoethanol, ≥99%) to isolate the protein. We homogenized the protein lysates and ran 10 μl of each in Western blots, and incubated the blots with the mouse monoclonal NR2B primary antibody (1:500, Thermo Scientific, Cat. #MA1-2014) overnight at 4 °C or with Actin controls in separate blots (Anti-actin monoclonal antibody, Millipore), followed by a secondary antibody CF^TM^680 labeled Goat Anti Mouse IgG1 (1:5000, Biotium 20065) for 2 hr at room temperature. All western blots were performed under the same experimental condition and the blots quantified using ImageJ.

### Subjects and housing

We collected 41 adult male zebra finches (~120–180 days old) and 4 30-day old juvenile birds (3 males and 1 female) from our breeding colony at Duke University to conduct pilot studies to test the lentivirus, and for final experimental manipulations. We housed the birds in cages in groups of four inside sound-attenuation recording chambers, provided them with food and water *ad libitum*, and maintained a constant photoperiod of 12:12 h light/dark cycle. We assigned 26 birds for pilot studies and housed the remaining birds for further manipulations later on. All animal procedures were approved by the Duke University Institutional Animal Care and Use Committee (IACUC), and all methods carried out in accordance with the guidelines and regulations of the IACUC-approved protocol number A095-14-04.

### Lentivirus injections

We anaesthetized male zebra finches with isoflurane anesthesia (1–2%; flow 1 L/min) and placed them in a stereotaxic apparatus (Kopf Instruments, Tujunga, CA). We used stereotaxic coordinates and multiunit electrophysiological recordings of the higher spontaneous activity in LMAN compared to surrounding nidopallium to locate LMAN bilaterally: 4.2–4.8 mm rostrally, 1.6–1.8 mm laterally, 2.2–2.4 mm ventrally. Once found, we injected the eGFPhNR2B or the control eGFP lentivirus (32.2 nl per injection totaling a final volume of ~1.2 μl) into LMAN using a glass micropipette attached to a pressure injection unit (Drummond Nanoinject II, Drummond Scientific Company, Broomall, PA). For a control brain region, we injected the lentiviral constructs in another set of animals into the nidopallium region adjacent to LMAN, using the following coordinates: 2.25 mm rostrally, 1.7 mm laterally, 2.2–2.3 mm ventrally. After surgery, we allowed the birds to awake and isolated them in a cage under a heat lamp with food and water for 48 hr before returning them to their original housing cage inside the sound attenuation boxes. To check for GFP expression in LMAN, we sacrificed a subset of birds injected with the eGFPhNR2B lentivirus (n = 3), or eGFP lentivirus (n = 3), 2.5 weeks after injections and found strong labeling of GFP in LMAN neurons indicating that both lentiviruses were able to infect LMAN.

### Microdissection and Western blot analyses of song nuclei to verify overexpression of *NR2B* protein

To validate overexpression of the NR2B protein within LMAN and to compare the levels with 30-day old juvenile birds, we performed Western blot analyses on brain tissue collected from a pilot set of adult birds (n = 7) that were injected with the eGFPhNR2B lentivirus, juvenile birds as mentioned above (n = 4), and from adult control birds with no injections (n = 11). At ~35–40 days post-lentiviral injections, we removed birds housed in recording chambers and immediately decapitated them. We quickly dissected and rinsed the brains in chilled PBS, and then mounted them in a tissue slicer (Stoelting Tissue Slicer, Cat. #51415, Wood Dale, IL, USA). Following the protocol described by Whitney *et al*.^50^, we first used a razor to cut off part of the brainstem to create a flat surface with the forebrain on a tissue block cutting surface. We positioned the cerebellum region against a thin plexiglass wall with Vetbond 3 M tissue adhesive on the surface of the tissue slicer. We cut 500 μm slices of the brain tissue in the coronal plane, starting from the front of the brain. The tissue slices were placed in a Petri dish (kept on ice) containing cold 0.1 M PBS that contained a protease inhibitor cocktail (cOmplete ULTRA Tablets, Cat. No. 06538304001; Roche, Indianapolis, IN, USA). We placed the Petri dish under a dissecting microscope and using fine surgical scissors (Cat. No. 15000-00; Fine Science Tools, Foster City, USA) and forceps (Cat. No. 11252-00; Fine Science Tools), we identified sections with song nuclei and quickly dissected out LMAN, Area X, and an adjacent nidopallium area of comparable size of LMAN from both hemispheres using a darkfield setting on an Olympus MVX10 microscope (Olympus, Japan). We were easily able to localize the song nuclei due to their denser neural fibers (white) observed in the darkfield setting. The dissected tissue was immediately frozen in microcentrifuge tubes placed in a dry ice-ethanol bath and then stored at −20 °C until further processing. Protein was isolated following SDS-PAGE 2X sample buffer as described before, and *NR2B* protein levels were detected using Western blot analysis using methods described previously.

### Song recordings and analyses

To assess the effect of manipulations on singing behavior, we recorded courtship (directed) songs from a set of birds (n = 17) that had been acclimatized in sound boxes for 2–4 days, at which time most birds sang at a normal singing rate of ~30–40 bouts of songs within the first one hour of singing. We focused on directed songs which are more stereotyped (less variable) and which males sing more intensely as opposed to undirected songs^90^. We inadvertently did not record sufficient amounts of undirected songs before surgery (due to too few minutes of recordings). However, we don’t expect results to be dramatically different with undirected songs, in that they tend to be more variable than directed songs, and our hypothesis was to specifically test for inducing song variability with *NR2B* overexpression. We recorded songs from each male for three weeks before surgical manipulations to capture the within-individual variation in their songs since males can vary their songs from one rendition to another rapidly. We define songs as a sequence of syllables that start with one or more introductory notes, continued with at least one or more motifs, and are surrounded by more than 2 seconds of silence. We stimulated directed songs by frequent switching every 5–10 minutes of presenting two female birds in the male cages in the morning for up to 1 hour, in which it is shown that the males almost exclusively produce directed songs^20, 91^. We used animals that sang more than 30 bouts of song per hour and produced short bursts of intense singing behavior that indicate courtship singing behavior. We recorded the songs with microphones (model SR0, Earthworks, Milford, NH, USA) that had a flat frequency response up to 20 kHz, connected to a computer with Avisoft RECORDER (www.avisoft.com) software at 16 bits and 44-kHz sampling rate. After we had collected enough presurgery directed song recordings, to characterize their song syllables within song motifs we assigned birds randomly to each of three surgery groups for lentiviral gene manipulations: (1) Birds that received injections of the eGFP lentivirus in LMAN (control group); (2) Birds that received injections of the eGFPhNR2B lentivirus in nidopallium (control group); and (3) Birds that received injections of the eGFPhNR2B lentivirus in LMAN (NR2B group). After surgery, we allowed the birds to recover for 2–3 days before recording their songs again for up to 2.5 months postsurgery. Birds were housed in groups of the same four individuals they were housed with before surgery, and their songs recorded one day every other week. We also ensured that the songs were selected and processed blind to the treatment group.

We analyzed the song syllable sequencing and acoustic features using Luscinia^92^, Sound Analysis Pro (SA+)^93, 94^, or a custom designed MATLAB software (MathWorks) developed by Dr. Masashi Tanaka^53^ based on the SVM algorithm^95^ generated by Dr. Kosuke Hamaguchi. For all acoustic analyses, we used 60 song bouts randomly chosen from each of both the presurgery, and postsurgery recordings of all individual males for a total of 120 songs for each bird. For each bird, we manually calculated the total syllable numbers within each motif, and number of introductory notes per motif, both before and after manipulations using Luscinia, and then averaged the scores for presurgery and postsurgery conditions. Using Sound Analysis Pro we computed the means of syllable or sequence similarity scores, and their associated song syllable acoustic features, including entropy, mean change in fundamental frequency, pitch, goodness of pitch, amplitude modulation, and their variances. These scores were averaged across all 60 song motifs for each of the presurgery and postsurgery songs. The three song similarity measurements included: (1) percentage similarity, that computes the threshold level of similarity between songs; (2) percentage of song syllable accuracy, that indicates the average local similarity score; and (3) sequential match, which computes the extent to which similar syllables across songs correspond in temporal order. Using the MathWorks program, we computed the means of intersyllable gaps of all syllables, syllable duration of all syllables, and song bout duration of song motifs. To compare the sequencing features before and after lentiviral injections in the control and NR2B groups, we calculated mean syllable sequence linearity, consistency, and stereotypy scores from the 120 song motifs using MathWorks as described in Tanaka *et al*.^53^. We calculated linearity as internal linearity^55^ (number of syllable types −1/number of transition types) with syllables including the introductory notes, but where repetition of introductory notes (such as iiiii) was excluded so that the syllable sequence “iiiiiabcd” produces the linearity score of 1. We calculated the consistency score as (number of typical transitions/number of transitions), where typical transitions were determined for each preceding syllable as the most frequently observed syllable transitions in the song. The stereotypy score was the average of linearity and consistency scores.

### Tissue collection, sectioning, and immunocytochemical detection of NR2B protein levels in LMAN

After their last recording session around 2.5 months after surgery, we removed the birds from the recording boxes, decapitated them, dissected and rinsed the brains in 0.1 M PBS within minutes, embedded the brain tissue in Tissue-Tek OCT Compound (Sakura, Finetek, Torrance, CA) in a plastic block mold and quickly froze the Tissue Tek around the brain on a dry ice-ethanol bath. The brain was stored at −80 °C until further processing. Brains were sectioned in sagittal planes at 16 μm in 6–20 series on a cryostat and the sections mounted on plus charged slides (Fisher Scientific, Pittsburgh, PA, USA), and again stored at −80 °C until further processing for immunohistochemistry.

To identify overexpression of NR2B protein, sections mounted on slides were fixed in 4% paraformaldehyde for 10 min, washed 5 X in 0.1 M PBS for 5 min each, and then incubated in 1% H_2_O_2_ in 0.1 M PBS for 30 min. After three 5 min washes in 0.1 M PBS, sections were incubated in 2% normal goat serum (NGS) in 0.1 M PBS with the addition of 0.2% Triton X-100 for 1 hr to prevent non-specific binding. The sections were then incubated for 72 hr with a primary antibody rabbit polyclonal anti-NR2B (Abcam, Cambridge, MA, USA), diluted 1:500 in PBS-T-N (0.2% Triton X-100 + 2% NGS) at 4 °C. The specificity of this antibody has been tested before in other avian species (Dr. Melvin Rouse, University of California, San Diego, *personal communication*) but we modified the protocol for use in zebra finches. After three 5 min washes in 0.2% PBS-T, the sections were incubated for 2 hr with a biotinylated goat anti-rabbit IgG antibody (Vector Laboratories Inc., Burlingame, CA, USA) at a dilution of 1:250 in PBS-T. After three 5 min washes in PBS-T, the sections were incubated in avidin-biotin horseradish-peroxidase complex (Vectastain Elite ABC kit Standard, PK-6100, Vector Laboratories) at a dilution of 1:200 in PBS-T for 1 hr. The sections were then washed twice for 5 min in 0.2% PBS-T, once in 0.1 M PBS, and then incubated in DAB buffer solution (SigmaFast DAB tablets, Sigma-Aldrich, St. Louis, MO) for 25 min. After five to six 5-min washes in 0.1 M PBS and one 5-min wash in deionized water, the slides were coverslipped with Vectashield Mounting medium with DAPI and allowed to dry for several days before microscopy.

We used brightfield microscopy on an Olympus MVX10 microscope to capture images of serial brain sections that spanned rostro-caudally (8–16 sections) containing the LMAN song nucleus from all subjects. To quantify the density of positive immuno-labeled cells, we developed a custom semi-automated Macro written in the Fiji (ImageJ) language that computes the number of stained cells in an image based on a darkness threshold selected by the user. We created a binary (black or white) mask of cells, determined a threshold for each image and counted labeled cells with the built in Particle Analyzer function in Fiji. The person quantifying the labeled cells using the macro was blind to the treatment groups of all animals.

### Statistical Analyses

All statistical analyses were performed in R (R Foundation for Statistical Computing, Vienna, Austria) and in SPSS (IBM SPPS Statistics for Macintosh, Version 23.0). When examining group means of both control and NR2B groups for pre- and post-surgery differences in song acoustic features we performed separate nonparametric paired Wilcoxon signed-rank tests for each comparison using the “wilcox.test (paired = TRUE)” function in R. To examine variability of song acoustic features, we computed the standard deviation (SD), and the coefficient of variation (c.v.) expressed as a percentage and used paired Wilcoxon signed-rank tests in R to test for statistical significance. To analyze if NR2B protein levels were correlated with song acoustic features we used linear regression models in SPSS. To analyze the stuttering effect and end syllable repetitions in the NR2B group from presurgery levels to different weeks after injections we used paired t tests in R. To analyze the NR2B protein levels between groups we used two sample Wilcoxon tests in R.

## Electronic supplementary material

Supplementary Information
