# Supplementary material for: Overexpression of human NR2B receptor subunit in LMAN causes stuttering and song sequence changes in adult zebra finches
Source: Sci Rep. 2017 Apr 21;7:942. doi: 10.1038/s41598-017-00519-8 (PMC5430713; doi:10.1038/s41598-017-00519-8)
Supplement: Supplementary file 1 — Supplementary Information [file 41598_2017_519_MOESM1_ESM.pdf]

Supplementary Information

Overexpression of human NR2B receptor subunit in LMAN causes stuttering and song sequence changes in adult zebra finches

Mukta Chakraborty*[1,2], Liang-Fu Chen+[1] , Emma E. Fridel+[1], Marguerita E. Klein[3], Rebecca Senft[1,2,4], Abhra Sarkar[5], Erich D. Jarvis*[1,2,6]

Supplementary Figure S1. Song acoustic features of motifs do not show changes from presurgery to postsurgery levels in the control group, but pitch differences are present in the NR2B group with other features remaining unchanged. Each line represents means (±SE) of one animal presurgery and postsurgery, with p values determined using paired Wilcoxon signed-rank tests.

Supplementary Figure S2. Variability (S.D.) of song acoustic features show no significant changes from presurgery to postsurgery levels in control or NR2B animals. Paired Wilcoxon signed-rank tests were conducted to test for changes in variability in both groups.

Supplementary Figure S3. Variability (S.D.) of song acoustic features and song similarity features show no significant changes from presurgery to postsurgery levels in control or NR2B animals. Paired Wilcoxon signed-rank tests were conducted to test for changes in variability in both groups.

Supplementary Figure S4. Variability expressed as percentage of coefficient of variation (% c.v.) of song acoustic features and song motif structure do not show changes from presurgery to postsurgery levels. Paired Wilcoxon signed-rank tests were conducted to test for changes in variability in both groups. Data are shown as mean (±SE).

Supplementary Figure S5. Regression plots of mean NR2B protein levels in LMAN with song and syllable features in animals injected with the eGFPhNR2B lentivirus in LMAN. Red boxes represent the control group mean that were not included in the regression analyses and are shown for comparison only.

Supplementary Figure S6. Regression plots of mean NR2B protein levels in LMAN with variability (S.D.) of song features in animals injected with the eGFPhNR2B lentivirus in LMAN. Red boxes represent the control group mean that were not included in the regression analyses and are shown for comparison only.

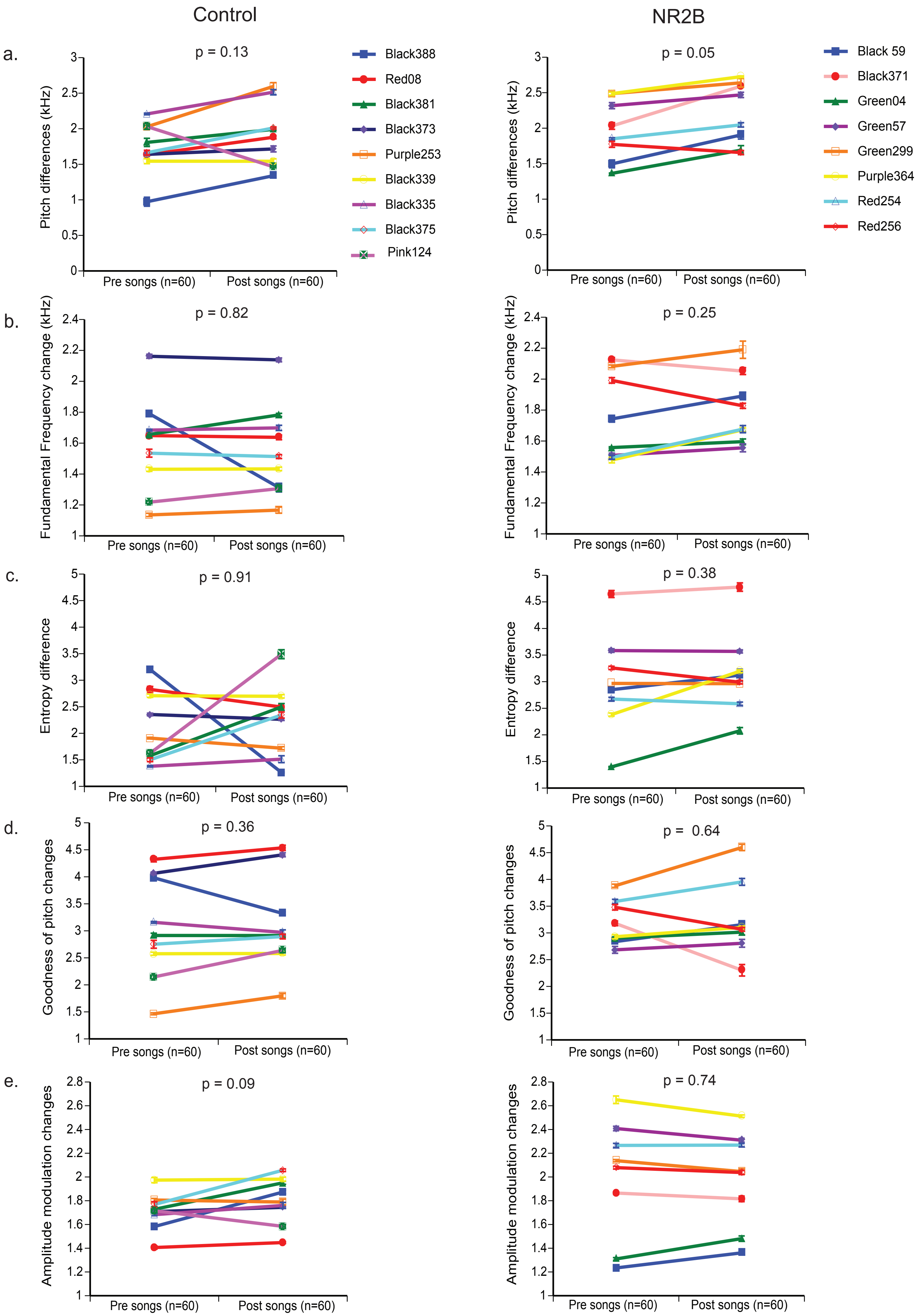

Control
NR2B
a.
b.
c.
d.
e.
p = 0.13
p = 0.05
p = 0.82
p = 0.25
p = 0.91
p = 0.38
p = 0.36
p = 0.64
p = 0.09
p = 0.74
Pitch differences (kHz)
Fundamental Frequency change (kHz)
Entropy difference
Goodness of pitch changes
Amplitude modulation changes
Pre songs (n=60)
Post songs (n=60)
Black388
Red08
Black381
Black373
Purple253
Black339
Black335
Black375
Pink124
Black 59
Black371
Green04
Green57
Green299
Purple364
Red254
Red256

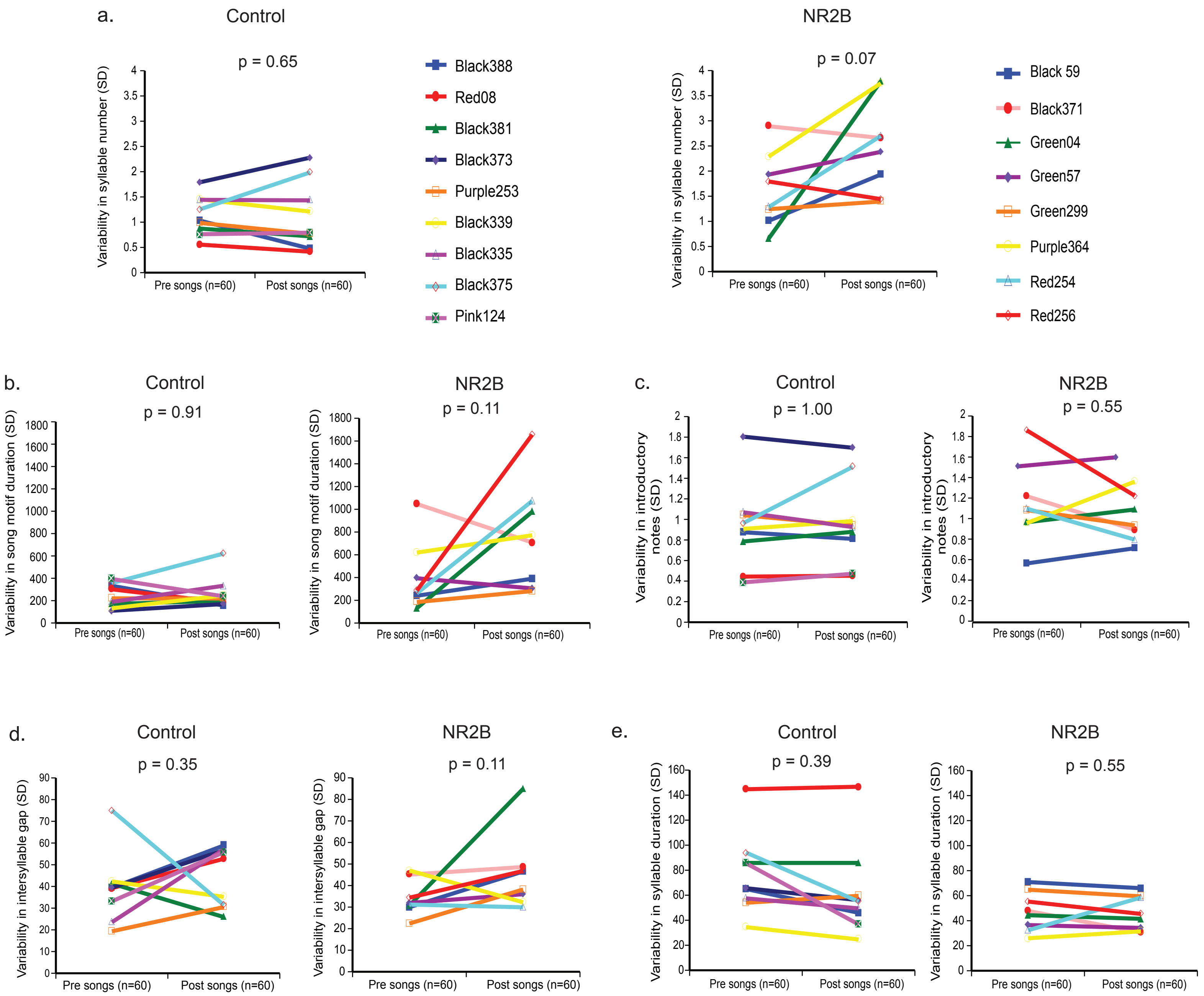
a.
Control
p = 0.65
Variability in syllable number (SD)
Pre songs (n=60)
Post songs (n=60)
Black388
Red08
Black381
Black373
Purple253
Black339
Black335
Black375
Pink124
NR2B
p = 0.07
Black 59
Black371
Green04
Green57
Green299
Purple364
Red254
Red256
b.
Control
p = 0.91
Variability in song motif duration (SD)
NR2B
p = 0.11
c.
Control
p = 1.00
Variability in introductory notes (SD)
NR2B
p = 0.55
d.
Control
p = 0.35
Variability in intersyllable gap (SD)
NR2B
p = 0.11
e.
Control
p = 0.39
Variability in syllable duration (SD)
NR2B
p = 0.55

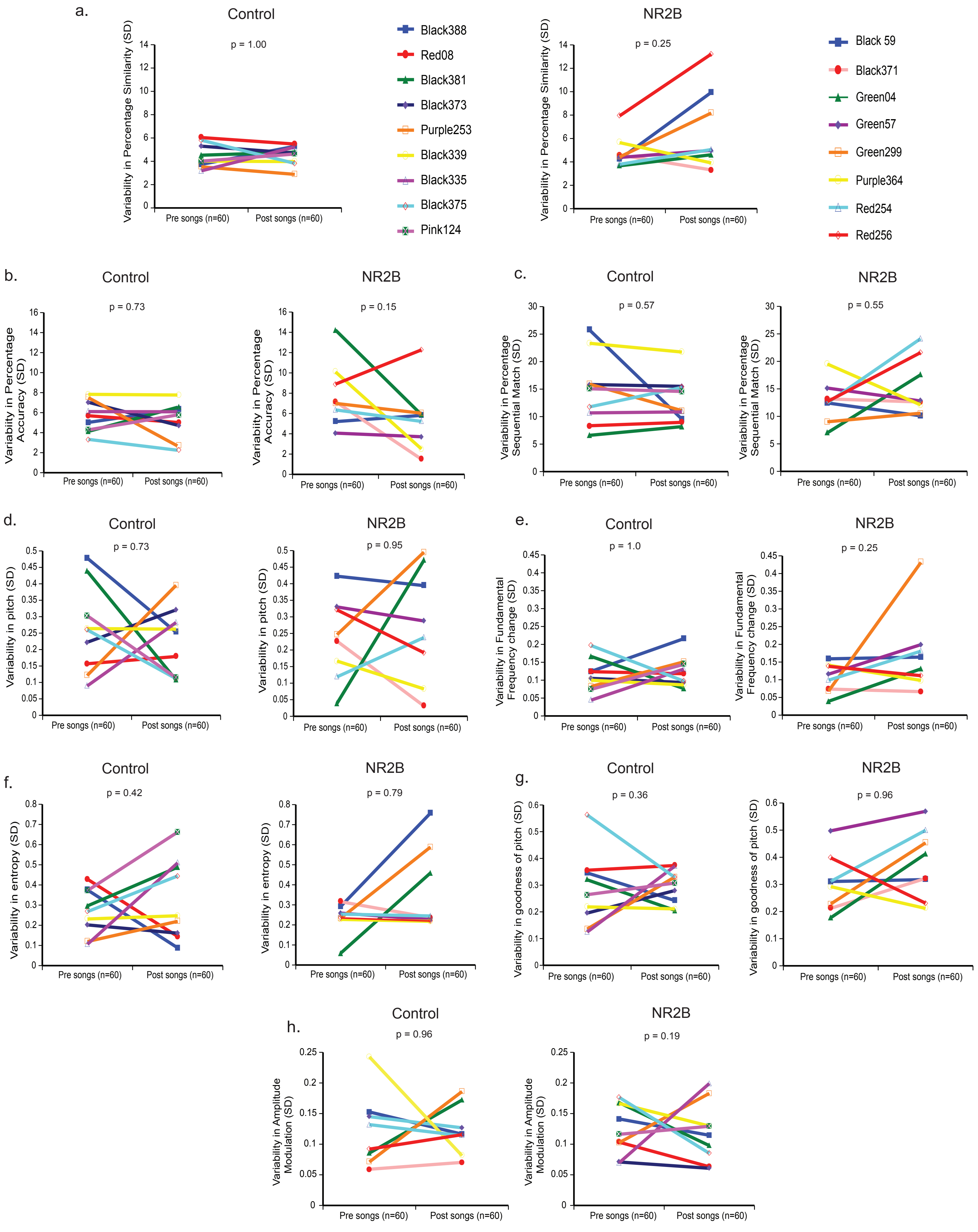
a.
Control
p = 1.00
Variability in Percentage Similarity (SD)
Pre songs (n=60)
Post songs (n=60)
Black388
Red08
Black381
Black373
Purple253
Black339
Black335
Black375
Pink124
NR2B
p = 0.25
Black 59
Black371
Green04
Green57
Green299
Purple364
Red254
Red256
b.
Control
p = 0.73
Variability in Percentage Accuracy (SD)
NR2B
p = 0.15
c.
Control
p = 0.57
Variability in Percentage Sequential Match (SD)
NR2B
p = 0.55
d.
Control
p = 0.73
Variability in pitch (SD)
NR2B
p = 0.95
e.
Control
p = 1.0
Variability in Fundamental Frequency change (SD)
NR2B
p = 0.25
f.
Control
p = 0.42
Variability in entropy (SD)
NR2B
p = 0.79
g.
Control
p = 0.36
Variability in goodness of pitch (SD)
NR2B
p = 0.96
h.
Control
p = 0.96
Variability in Amplitude Modulation (SD)
NR2B
p = 0.19

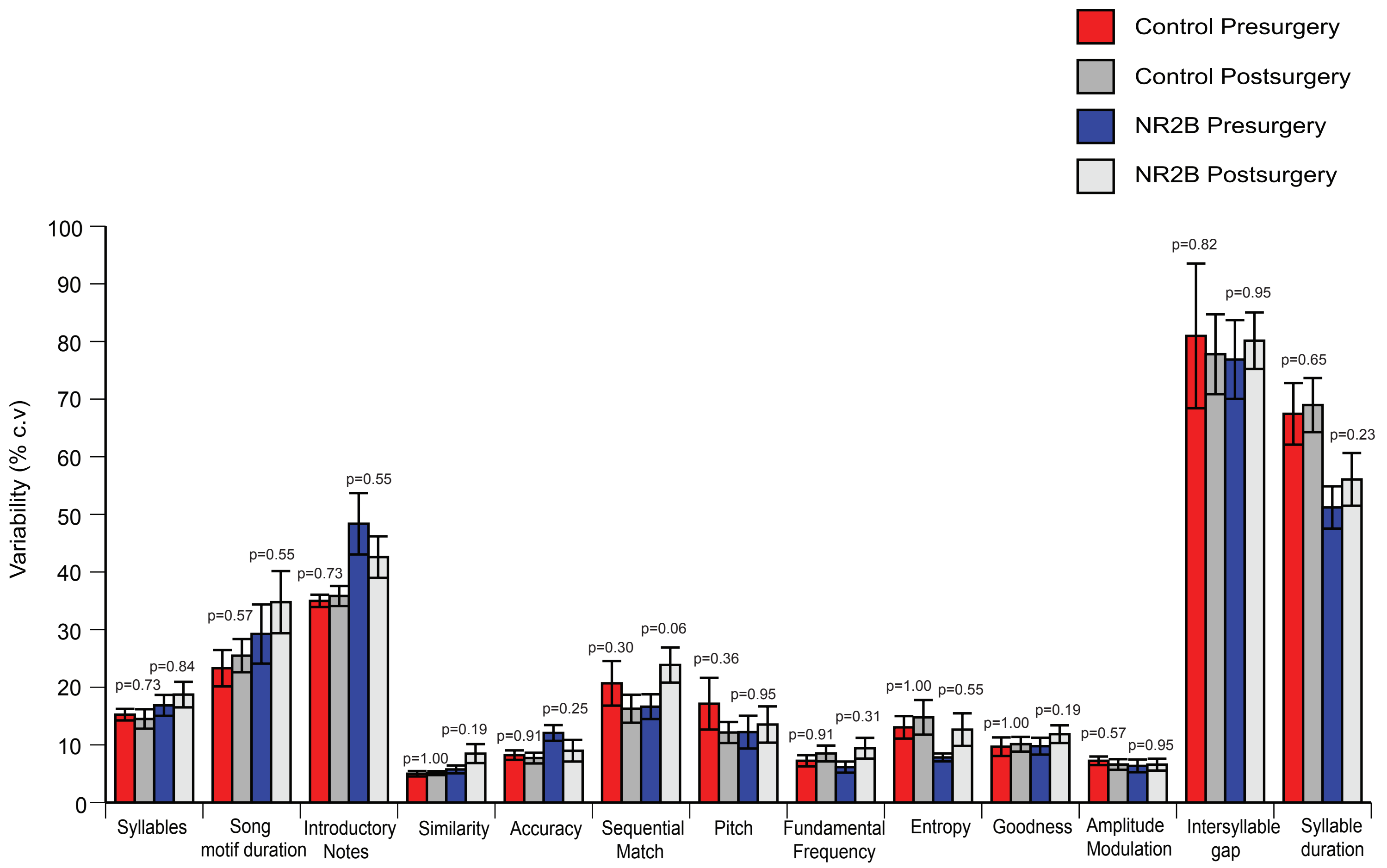

Control Presurgery
Control Postsurgery
NR2B Presurgery
NR2B Postsurgery
Variability (% c.v)
100
90
80
70
60
50
40
30
20
10
0
p=0.73
p=0.84
p=0.57
p=0.55
p=0.73
p=0.55
p=1.00
p=0.19
p=0.91
p=0.25
p=0.30
p=0.06
p=0.36
p=0.95
p=0.91
p=0.31
p=1.00
p=0.55
p=1.00
p=0.19
p=0.57
p=0.95
p=0.82
p=0.95
p=0.65
p=0.23
Syllables
Song motif duration
Introductory Notes
Similarity
Accuracy
Sequential Match
Pitch
Fundamental Frequency
Entropy
Goodness
Amplitude Modulation
Intersyllable gap
Syllable duration

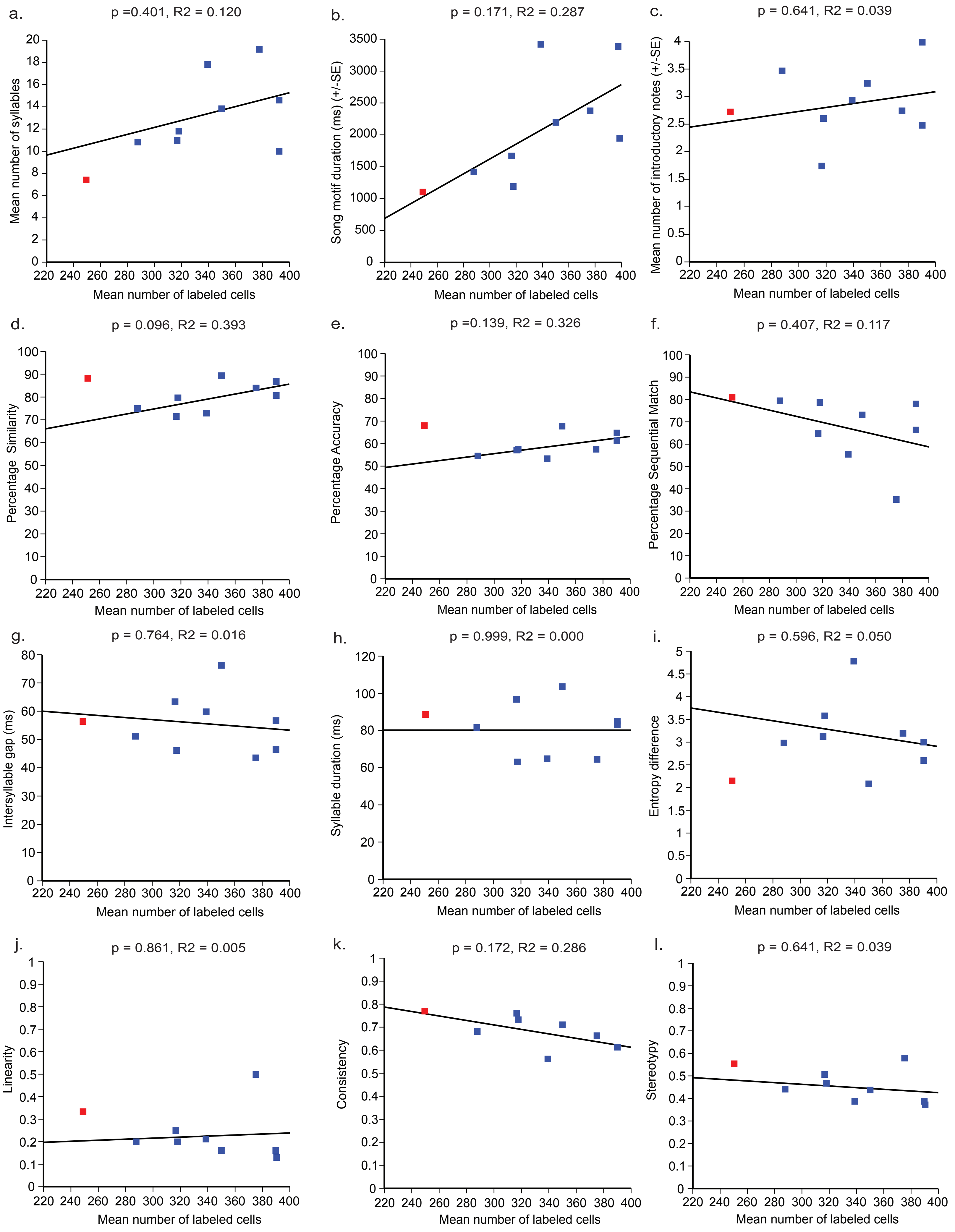
a.
p =0.401, R2 = 0.120
Mean number of syllables
Mean number of labeled cells
b.
p = 0.171, R2 = 0.287
Song motif duration (ms) (+/-SE)
Mean number of labeled cells
c.
p = 0.641, R2 = 0.039
Mean number of introductory notes (+/-SE)
Mean number of labeled cells
d.
p = 0.096, R2 = 0.393
Percentage Similarity
Mean number of labeled cells
e.
p =0.139, R2 = 0.326
Percentage Accuracy
Mean number of labeled cells
f.
p = 0.407, R2 = 0.117
Percentage Sequential Match
Mean number of labeled cells
g.
p = 0.764, R2 = 0.016
Intersyllable gap (ms)
Mean number of labeled cells
h.
p = 0.999, R2 = 0.000
Syllable duration (ms)
Mean number of labeled cells
i.
p = 0.596, R2 = 0.050
Entropy difference
Mean number of labeled cells
j.
p = 0.861, R2 = 0.005
Linearity
Mean number of labeled cells
k.
p = 0.172, R2 = 0.286
Consistency
Mean number of labeled cells
l.
p = 0.641, R2 = 0.039
Stereotypy
Mean number of labeled cells

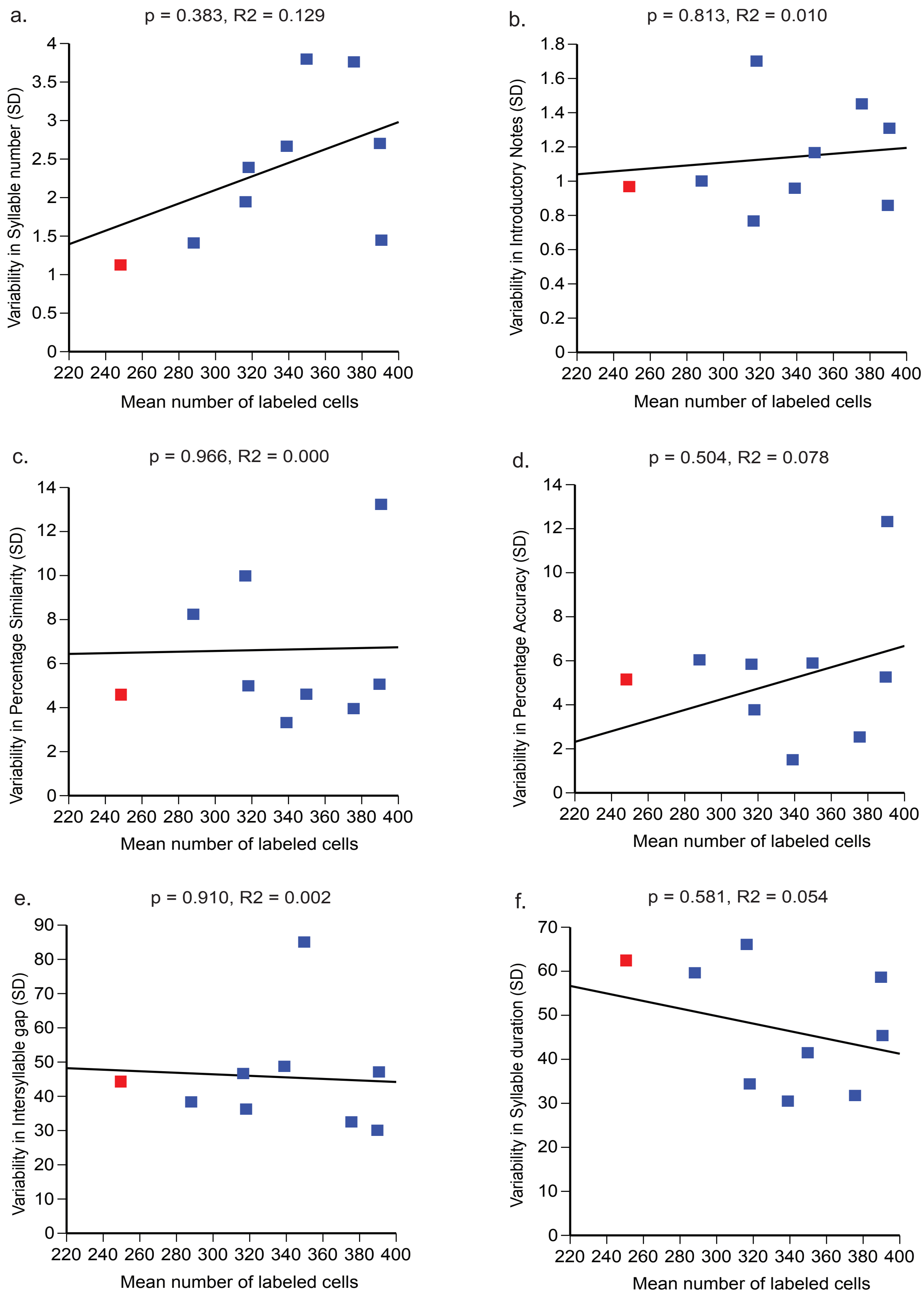
a.
p = 0.383, R2 = 0.129
Variability in Syllable number (SD)
Mean number of labeled cells
b.
p = 0.813, R2 = 0.010
Variability in Introductory Notes (SD)
Mean number of labeled cells
c.
p = 0.966, R2 = 0.000
Variability in Percentage Similarity (SD)
Mean number of labeled cells
d.
p = 0.504, R2 = 0.078
Variability in Percentage Accuracy (SD)
Mean number of labeled cells
e.
p = 0.910, R2 = 0.002
Variability in Intersyllable gap (SD)
Mean number of labeled cells
f.
p = 0.581, R2 = 0.054
Variability in Syllable duration (SD)
Mean number of labeled cells
